# Ciliary dynein motor preassembly is regulated by Wdr92 in association with HSP90 co-chaperone, R2TP

**DOI:** 10.1083/jcb.201709026

**Published:** 2018-07-02

**Authors:** Petra zur Lage, Panagiota Stefanopoulou, Katarzyna Styczynska-Soczka, Niall Quinn, Girish Mali, Alex von Kriegsheim, Pleasantine Mill, Andrew P. Jarman

**Affiliations:** 1 Centre for Discovery Brain Sciences, Edinburgh Medical School, University of Edinburgh, Edinburgh, Scotland, UK; 2 Medical Research Council Human Genetics Unit, Institute of Genetics and Molecular Medicine, University of Edinburgh, Edinburgh, Scotland, UK; 3 Edinburgh Cancer Research UK Centre, Institute of Genetics and Molecular Medicine, University of Edinburgh, Edinburgh, Scotland, UK; 4 Systems Biology Ireland, University College Dublin, Belfield, Dublin, Ireland

## Abstract

The massive dynein motor complexes that drive ciliary and flagellar motility require cytoplasmic preassembly, a process requiring dedicated dynein assembly factors (DNAAFs). How DNAAFs interact with molecular chaperones to control dynein assembly is not clear. By analogy with the well-known multifunctional HSP90-associated cochaperone, R2TP, several DNAAFs have been suggested to perform novel R2TP-like functions. However, the involvement of R2TP itself (canonical R2TP) in dynein assembly remains unclear. Here we show that in *Drosophila melanogaster*, the R2TP-associated factor, Wdr92, is required exclusively for axonemal dynein assembly, likely in association with canonical R2TP. Proteomic analyses suggest that in addition to being a regulator of R2TP chaperoning activity, Wdr92 works with the DNAAF Spag1 at a distinct stage in dynein preassembly. Wdr92/R2TP function is likely distinct from that of the DNAAFs proposed to form dynein-specific R2TP-like complexes. Our findings thus establish a connection between dynein assembly and a core multifunctional cochaperone.

## Abstract (precis)

Wdr92 is associated with the multifunctional cochaperone, R2TP, but its function is unknown. In this study, the authors show that *Drosophila* Wdr92 is exclusively required for preassembly of ciliary dynein motor complexes, which are confined to sensory neuron ciliary dendrites and sperm flagella. Wdr92 is proposed to direct R2TP/HSP90 to dynein chain clients to chaperone cytoplasmic preassembly.

## Introduction

Many tasks within the cell are accomplished by highly organized multicomponent protein complexes, the biogenesis of which requires guided assembly by molecular chaperones (Makhnevych and Houry, 2012). Particularly striking examples are the axonemal dynein motor complexes of motile cilia. These massive multisubunit complexes are visible by electron microscopy as outer and inner dynein arms (ODA and IDA, respectively; King, 2016). The motors require a complex pathway of preassembly in the cytoplasm before their transport to the cilium (Fok et al., 1994; Fowkes and Mitchell, 1998; Mitchison et al., 2012). In recent years, a group of some 10 proteins have been identified that facilitate this assembly pathway—collectively known as axonemal dynein assembly factors (DNAAFs; Mitchison et al., 2012). Several of these proteins were discovered through identifying causative mutations in human primary ciliary dyskinesia (PCD), characterized by ciliary/flagellar immotility (Omran et al., 2008; Mitchison et al., 2012; Knowles et al., 2013; Moore et al., 2013; Tarkar et al., 2013; Zariwala et al., 2013; Diggle et al., 2014). These assembly factors are largely cytoplasmic, and when their function is impaired, ODAs and/or IDAs are missing from the ciliary axoneme. Although the specific functions of most of these assembly factors are unclear, based on protein sequence and protein interactions, several appear to recruit the ubiquitous chaperone, HSP90. HSP90 functions at late stages of protein folding (Wandinger et al., 2008; Taipale et al., 2010), and is particularly associated with the assembly of protein complexes (Boulon et al., 2010). It has a wide range of potential clients, although few have been extensively analyzed in vivo (Zhao et al., 2005; McClellan et al., 2007). Aside from PCD, protein homeostasis is increasingly associated with diseases from neurodegeneration to cancer, and is a promising target for therapeutic intervention (Lindquist and Kelly, 2011).

In *Drosophila melanogaster*, the entire repertoire of known DNAAFs is conserved (unpublished data), but the fly has only two cell types bearing cilia with motile characteristics: the sperm flagellum and the ciliated sensory dendrite of auditory/proprioceptive chordotonal (Ch) sensory neurons (Cachero et al., 2011), in which ciliary motility seems to be critical in mechanosensory transduction (Karak et al., 2015). Therefore, mutations of DNAAF genes specifically result in auditory/proprioceptive deficiency and male infertility (Kavlie et al., 2010; Moore et al., 2013; Diggle et al., 2014). The restricted expression and function of motile cilium genes in *Drosophila* accelerates discovery and characterization of new motility-related genes: a screen for such genes led to the discovery of PCD-causative genes, *ZMYND10* and *DNAAF5/HEATR2* (Moore et al., 2013; Diggle et al., 2014). In the same screen, we identified *CG14353*, which encodes a homologue of the human WD40-repeat protein, WDR92 (also known as MONAD; *CG14353* is hereafter referred to as *Wdr92*). WDR92 is poorly characterized functionally, although overexpression studies in cell culture suggested a link to apoptosis (Saeki et al., 2006). WDR92 is known best from several proteomic studies through its binding to the RPAP3 subunit of the R2TP cochaperone complex (Sardiu et al., 2008; Glatter et al., 2011; Cloutier et al., 2017), which is potentially of great significance for dynein preassembly. R2TP brings clients to the chaperones HSP70/HSP90 in the assembly and/or stabilization of a variety of protein complexes from yeast to human (Boulon et al., 2010, 2012), including small nuclear RNA–protein complexes (snoRNPs), phosphatidylinositol-3 kinase-related protein kinase (PIKK)–containing complexes (e.g., mTOR), and RNA polymerases (Kakihara and Houry, 2012; von Morgen et al., 2015). Although disruption of the R2TP-associated helicases *Reptin* (*Ruvbl2*) or *Pontin* (*Ruvbl1*) causes phenotypes associated with impaired cilia motility in zebrafish (Zhao et al., 2013), this has been interpreted as reflecting their participation with certain DNAAFs in cilium-specific alternative R2TP-like complexes (Tarkar et al., 2013; Pal et al., 2014; Vaughan, 2014; Li et al., 2017; Olcese et al., 2017; Paff et al., 2017). In contrast, “canonical” R2TP itself has not been directly linked to dynein assembly. In addition, in proteomic analyses, WDR92 is consistently found associated with the prefoldin-like complex (Sardiu et al., 2008), which by analogy to the canonical prefoldin complex is proposed to have chaperone activity (Millán-Zambrano and Chávez, 2014). However, the functional significance of this association is unknown.

Comparative genomics demonstrate that *WDR92* genes are specifically associated with organisms that bear motile cilia (Baron et al., 2007), and indeed, recent research supports a role in planarian ciliogenesis, although its function was not defined (Patel-King and King, 2016). To test the hypothesis that Wdr92 (and R2TP) functions in dynein preassembly, we explored its function in *Drosophila*. We show that *Drosophila Wdr92* is a cytoplasmic protein exclusively expressed in motile ciliated cells and is required exclusively for ciliary/flagellar motility. The major effect of its mutation is loss of dynein arms from the axonemes of sensory neuron cilia and sperm flagella. We show that Wdr92 associates with the DNAAF Spag1, confirm that *Drosophila* Wdr92 also interacts with R2TP, and show that R2TP depletion also impairs dynein arm formation. We show that Wdr92 protein is associated with both dynein heavy chains (HCs) and intermediate chains (ICs), and propose that it acts as a specificity factor to bring partially assembled dynein clients to R2TP/HSP90 at a late stage of cytoplasmic assembly. Thus, *Drosophila Wdr92* is a new DNAAF that strongly reinforces the critical role of HSP90 and cochaperones in dynein assembly.

## Results

### *Drosophila Wdr92* is expressed in developing Ch neurons and sperm

**Figure 1.**
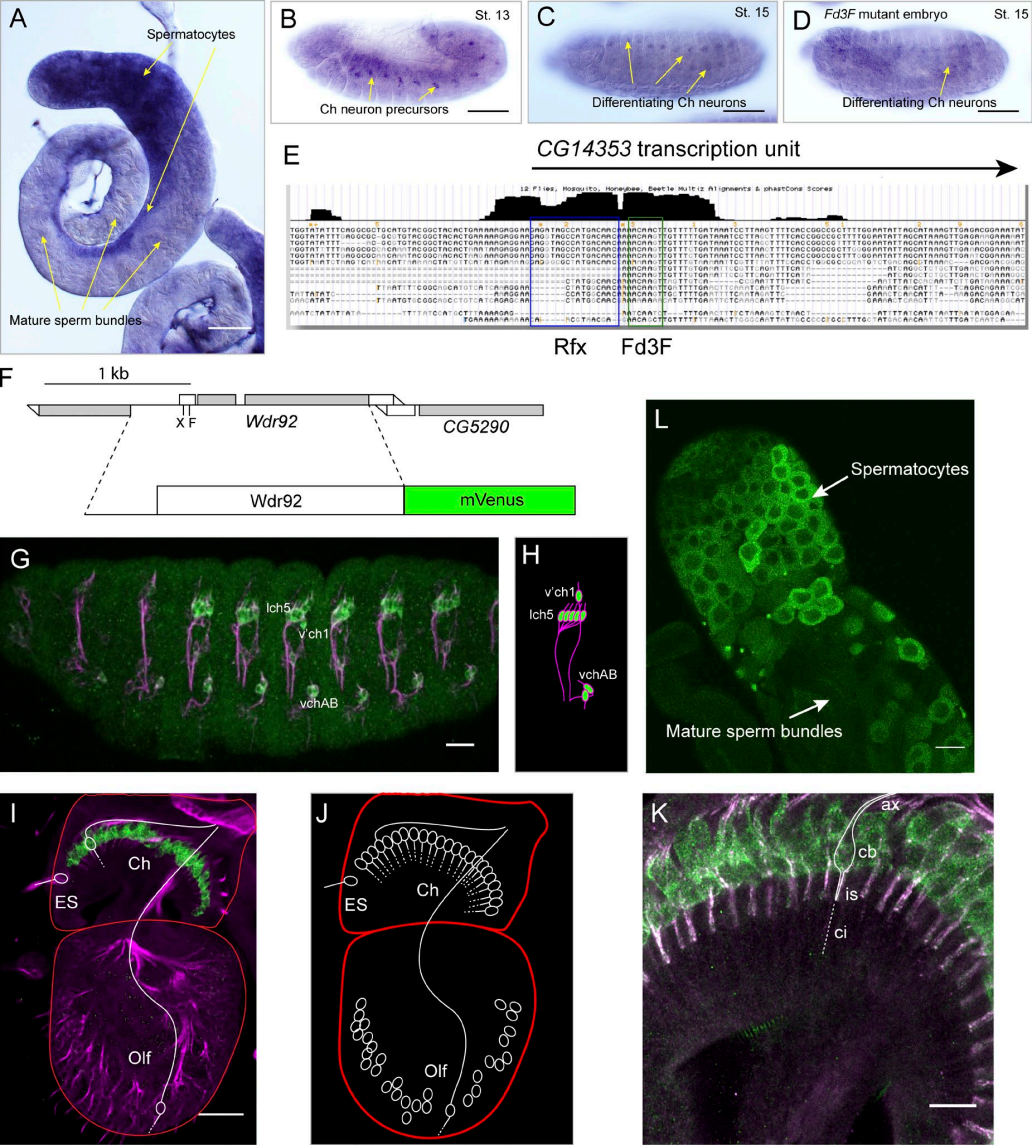
***Drosophila* Wdr92 protein is expressed exclusively in cytoplasm of motile ciliated cells. (A–D)** RNA in situ hybridization with *Wdr92* probe. **(A)** Adult testes, showing expression in spermatocytes, but not mature sperm. **(B)** Stage 13 embryo, showing expression in Ch neuron precursors. **(C)** Stage 15 embryo, showing expression in differentiating Ch neurons. **(D)** Stage 15 embryo mutant for transcription factor *Fd3F*, showing reduction in *Wdr92* expression in Ch neurons. **(E)** Screenshot from UCSC genome browser of alignment of insect sequences around the *Wdr92/CG14353* transcription start site, showing location of matches for Rfx and Fd3F DNA binding motifs. The black bars above represent the sequence conservation across insect species. **(F)** Schematic of Wdr92-mVenus fusion gene transgene construct. It includes the 5′ upstream region, 5′ UTR, and entire ORF of *Wdr92*. **(G–L)** Green: mVenus; magenta: sensory neuron marker, anti-Futsch. **(G)** Expression of Wdr92-mVenus (green) in cytoplasm of Ch neurons of late stage embryo. **(H)** Schematic of Ch neuron arrangement in embryonic abdominal segment. **(I)** Expression of Wdr92-mVenus (green) in cytoplasm of Ch neurons of Johnston’s organ in pupal antenna, but not external sensory (ES) or olfactory neurons (Olf; example neurons schematically outlined). **(J)** Schematic of the sensory neurons in the pupal antenna. **(K)** Higher magnification of pupal antennal Ch neurons (stained as in I), with one schematically outlined to show the location of cell bodies (cb), axon (ax), dendritic inner segment (is), and sensory cilium (ci, also known as the dendritic outer segment). **(L)** Adult testis, expression in cytoplasm of spermatocytes before flagellogenesis. Bars: (A–D) 100 µm; (G, I, and L) 30 µm; (K) 10 µm.

FlyAtlas adult expression data show that *Wdr92* is highly and specifically expressed in testes (Robinson et al., 2013). Testis expression is confirmed by RNA in situ hybridization, which shows expression in round spermatocytes but not mature sperm (Fig. 1 A). In embryos, *Wdr92* mRNA is present in differentiating Ch neurons and their precursors (Fig. 1, B and C). Moreover, this expression is strongly reduced in embryos bearing a mutation in *Fd3F*, which encodes a FOXJ1-orthologous transcription factor that regulates cilia motility genes in cooperation with the ciliogenic transcription factor, Rfx (Fig. 1 D; Cachero et al., 2011; Newton et al., 2012). Consistent with being an Fd3F/Rfx target gene, the *Wdr92* 5′UTR has a conserved pair of Rfx and Fd3F binding motifs common to other target genes (Fig. 1 E).

Expression exclusive to Ch neurons was confirmed in embryos expressing a *Wdr92-mVenus* fusion protein reporter (present as a transgene driven by the *Wdr92* promoter; Fig. 1, F–H). In the pupal antenna, *Wdr92-mVenus* expression was detected in the differentiating Ch neurons that comprise Johnston’s organ (required for hearing and proprioception), but not in other ciliated (but nonmotile) sensory neurons (Fig. 1, I–K). Fusion protein expression was also detected in the testis within developing spermatocytes but not in mature sperm (Fig. 1 L). In all cases, expression of the fusion protein was not observed in the cilium/flagellum but was confined to the cytoplasm. Thus, *Wdr92* is a cytoplasmic protein expressed during the development of the only two cell types that bear cilia/flagella with motile features.

### *Wdr92* is required for Ch neuron function and sperm motility

**Figure 2.**
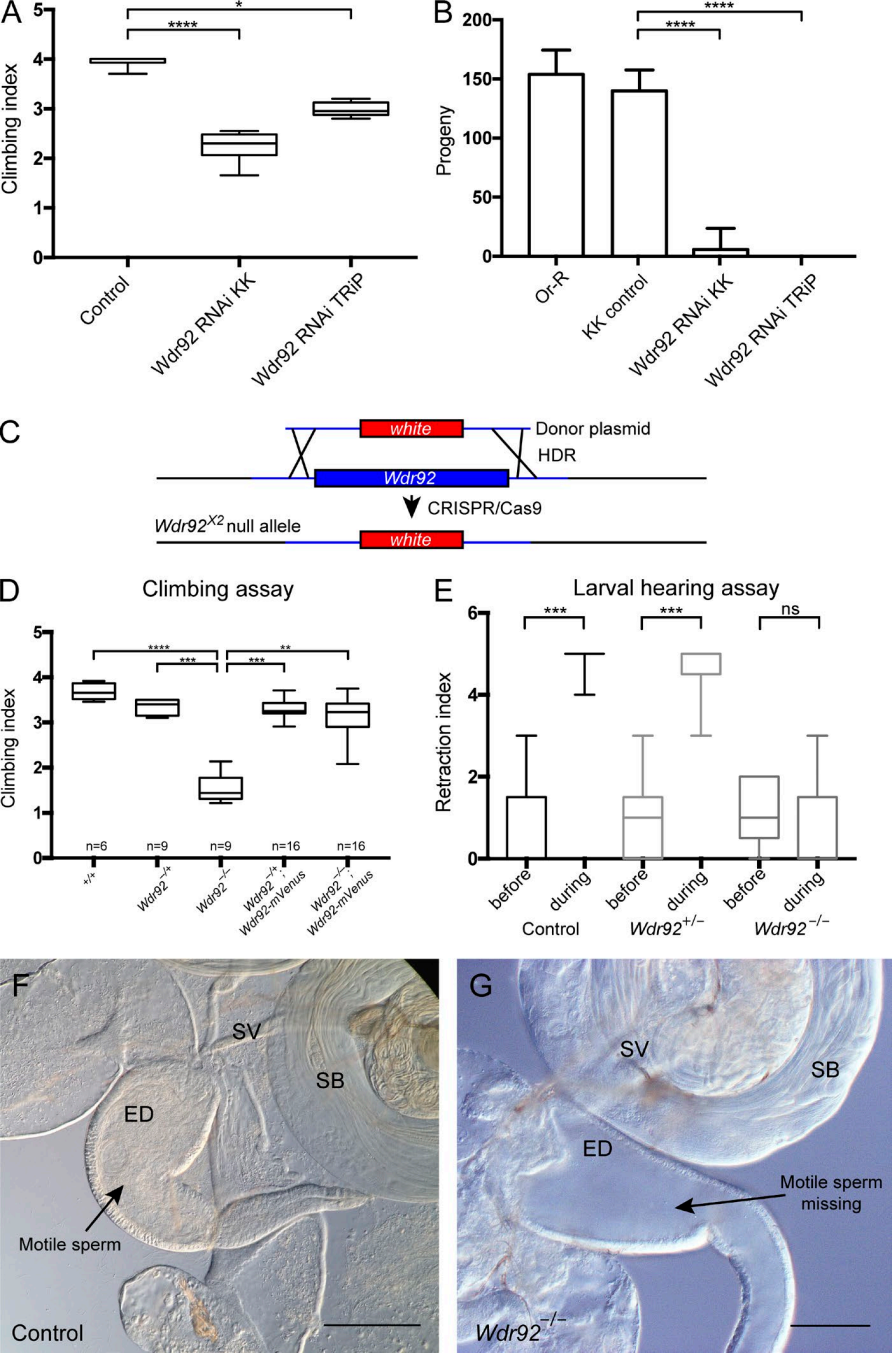
**Motile ciliated cells are functionally defective in *Wdr92* mutants. (A)** Box plot (median and interquartile range) of adult climbing assay for two independent *Wdr92* RNAi lines crossed to *sca*Gal4 driver (Table S4). Climbing index (proprioceptive performance) is reduced relative control flies (*sca*Gal4 crossed to KK parent line). *n* = 5 batches of 10–15 flies. **(B)** Fertility assay (mean and SD of progeny per male, *n* = 10 males) for *Wdr92* RNAi lines crossed to *Bam*Gal4 driver. Controls are males from KK parent line or OregonR (Or-R) crossed to *Bam*Gal4. **(C)** Schematic diagram of generation of *Wdr92* deletion mutation by ORF replacement with mini-*white* gene, mediated by CRISPR/Cas9 using homology-directed repair. **(D)** Adult climbing assay (median and interquartile range) of *Wdr92^−/−^* deletion mutant, including rescue by Wdr92-mVenus fusion gene. **(E)** Larval hearing assay. Retraction index (median and interquartile range) reflects the larval retraction response before and during a 1000 Hz tone. *n* = 9 batches of five larvae. **(F)** Control male reproductive organs, showing motile sperm within SV and ejaculatory duct (ED), and maturing sperm bundles (SB) in the testes. **(G)** *Wdr92^−/−^* mutant, showing sperm bundles, but lack of motile sperm in SVs or ED, consistent with sperm motility being required for transfer to these structures (Moore et al., 2013). Significance was determined by Kruskal-Wallis test, with Dunn’s test for multiple comparisons (A, D, and E) or ordinary one-way ANOVA, with Dunnett’s test for multiple comparisons (B). Significance on plots is signified by asterisks: *, P ≤ 0.05; **, P ≤ 0.01; ***, P ≤ 0.001; ****, P ≤ 0.0001. Bars, 100 µm.

In initial experiments, we depleted *Wdr92* expression using flies with two independent Gal4-inducible RNAi constructs. When depleted in developing Ch neurons (using a UAS-*Dcr2*; *scaGal4* driver line), resulting adult flies showed defective behavior in a climbing assay (Fig. 2 A). Combined with Ch-neuron–specific expression, this indicates defective proprioception resulting from impaired Ch neuron function in adult antennae and legs. Depletion in the male germline (*BamGal4*) resulted in complete infertility (*n* = 10 males; Fig. 2 B).

These phenotypes were confirmed in a null allele of *Wdr92* generated by CRISPR/Cas9 gene replacement of the entire ORF with a mini-*white* gene (*Wdr92^x2^*, henceforth referred to as *Wdr92^−/−^*; Fig. 2 C). *Wdr92^−/−^* flies were viable with no visible defects, indicating that *Wdr92* is not required for general cell viability. However, the flies exhibited poor proprioception in a climbing assay, and this was rescued by the *Wdr92-mVenus* fusion gene (Fig. 2 D). Moreover, the auditory function of larval body wall Ch neurons was defective in a behavioral assay for larval hearing (Fig. 2 E). *Wdr92^−/−^* males were completely infertile. Testes dissected from *Wdr92^−/−^* males had sperm bundles clearly visible, but no motile sperm were observed in the seminal vesicles (SVs), and none were released after crushing of the testes, in contrast to controls (*n* = 10 males; Fig. 2, F and G).

### *Wdr92* is required for the presence of axonemal dynein arms

**Figure 3.**
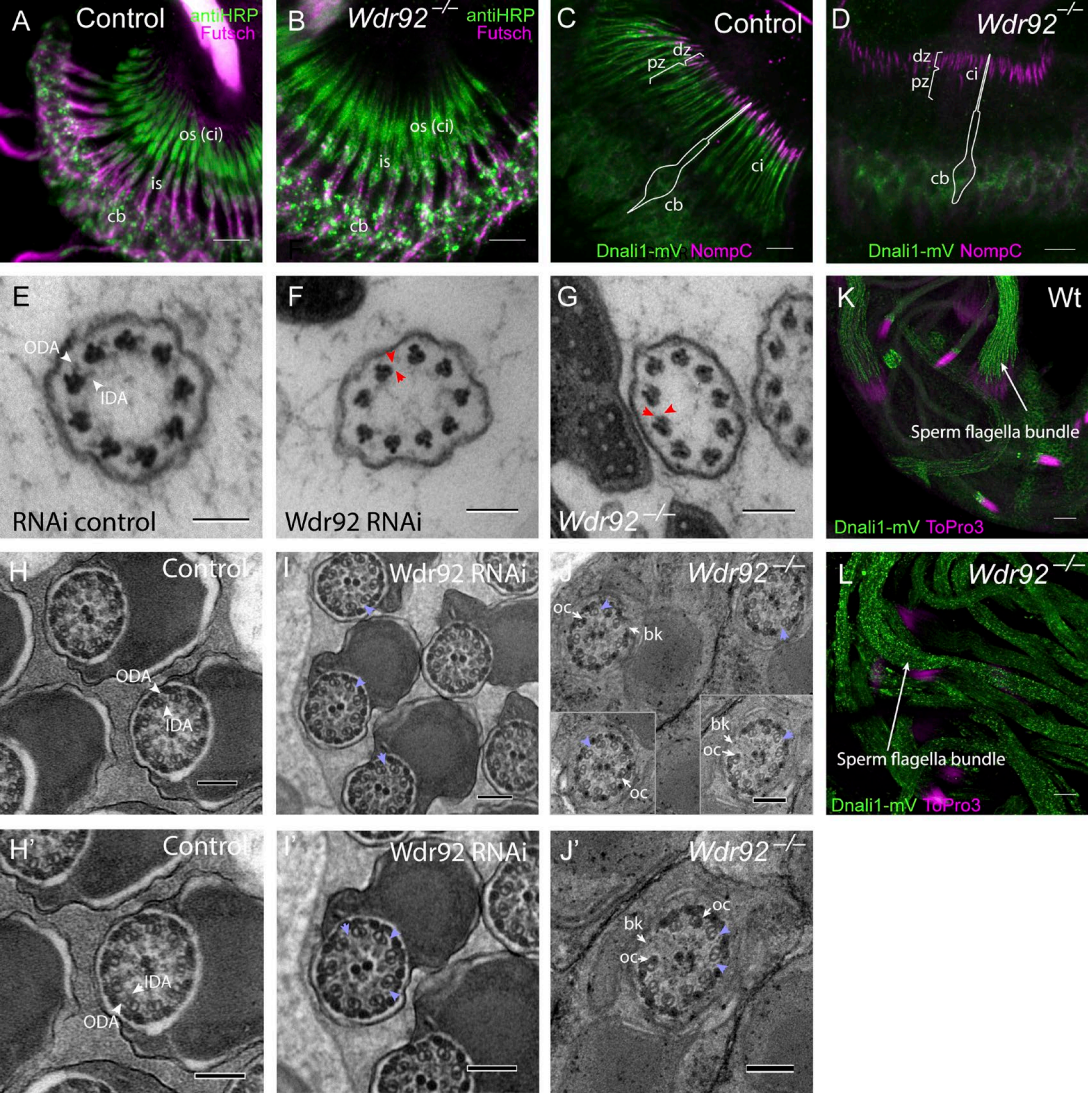
***Wdr92* is required for axonemal outer and IDAs. (A–D)** Pupal antenna. **(A)** Control with sensory neuron markers anti-HRP (green) and anti-Futsch (magenta; cb, cell bodies; is, inner segment of dendrites; os, outer segment of dendrites, which are ciliated, ci). **(B)** Similar localization of markers in *Wdr92* mutant Ch neurons. **(C)** Control, showing localization of NompC to distal zone (dz) of cilia (ci) and Dnali1-mVenus to proximal (motile) zone (pz). Schematic outline of a Ch neuron is indicated. **(D)** *Wdr92* mutant, showing lack of localization of Dnali1-mVenus to proximal zone. **(E–G)** TEM of adult antennal Ch neurons, transverse sections through the terminal sensory cilia. **(E)** Control showing 9+0 axoneme with ODAs and IDAs (white arrowheads). These are absent in otherwise normal cilia in *Wdr92* RNAi knockdown (F) and mutant (G; red arrowheads mark examples of expected ODA/IDA location). **(H–J)** TEM of testes, transverse sections through sperm bundles (with higher magnifications of these panels in H′–J′). **(H and H′)** Control, showing 9+2 axonemes and ODA/IDA. ODA/IDA are absent in RNAi knockdown (I and I′) and mutant (J and J′) testes (red arrowheads show examples). Also observed at lower frequency are breakage of axoneme (bk) and occluded A microtubules (oc). **(K and L)** Localization of Dnali1-mVenus (green) in adult testes. **(K)** Control adult testis showing localization along flagellar axonemes of sperm bundles, which consist of 64 aligned flagella (sperm nuclei labeled with ToPro, magenta). **(L)** *Wdr92* mutant adult testis, showing punctate aggregation of Dnali1-mVenus within the sperm bundles. Bars: (A–D) 5 µm; (E–J, including insets) 100 nm; (K and L) 10 µm.

Immunofluorescence analysis of *Wdr92^−/−^* embryos and pupal antennae revealed grossly normal Ch neuron structures, and the presence of the sensory cilium appeared unaffected (Fig. 3, A and B). Transmission electron microscopy (TEM) confirmed a grossly normal ultrastructure of the Ch neuron sensory cilia, with normal basal body, transition zone, ciliary dilation, and ciliary rootlet (unpublished data). However, transverse sections of the proximal cilium in both deletion and depletion mutants showed a complete absence of the ODA/IDA normally observed in this region (*Wdr92^−/−^*: 16/16 cilia showed complete loss of arms; *Wdr92^+/−^* heterozygote control: 14/14 cilia showed normal arms) (Fig. 3, E–G). To confirm this phenotype, we examined the localization of a dynein subunit, Dnali1/CG6971, using a Dnali1-mVenus line (Diggle et al., 2014). When IDA assembly occurs normally, Dnali1 is located in the proximal motile zone of Ch neuron cilia (pz in Fig. 3 C), but it was completely absent from *Wdr92^−/−^* cilia (Fig. 3 D). In contrast, the mechanosensory ion channel NompC was correctly localized in the distal (nonmotile) zone of the cilium (Fig. 3, C and D). Therefore, by ultrastructure and immunofluorescence analysis, *Wdr92* mutation specifically affects dynein motors. This contrasts with planarian *Wdr92*, the depletion of which resulted in diverse defects in ciliogenesis (Patel-King and King, 2016).

TEM analysis of sperm bundles in the adult testis also showed a lack of dynein arms in both *Wdr92* deletion and depletion mutant and testes (Fig. 3, H–J). Apart from this, sperm bundles seemed largely unaffected (with ∼64 spermatids per bundle), although sperm individualization may be impaired as was reported for a dynein chain mutation (Fatima, 2011). In addition to dynein arm loss, axonemal A microtubules were sometimes occluded, and individual microtubule doublet complexes sometimes separated from the rest of the axoneme (Fig. 3 J). These features had been previously noted for *Drosophila* homologues of several DNAAFs, notably *tilB/LRRC6* (Kavlie et al., 2010), *Zmynd10* (Moore et al., 2013), *Heatr2* (Diggle et al., 2014), and *Dnaaf3/CG17669* (unpublished data). Interestingly, occluded A tubules were also observed for planarian *Wdr92* knockdown (Patel-King and King, 2016). In wild-type testis, Dnali1-mVenus strongly localizes to the maturing flagellar axonemes in the sperm bundles (Fig. 3 K). This is disrupted in *Wdr92^−/−^* testes—Dnali1-mVenus protein is not localized along the flagella, but is present in the vicinity of the flagella in aggregates (Fig. 3 L). This difference in Dnali1-mVenus localization between sperm and Ch neurons might reflect differences in dynein transport during ciliogenesis: in Ch neuron ciliogenesis, transport is likely dependent on intraflagellar transport, but sperm flagellum synthesis proceeds within the cytoplasm in a non-intraflagellar transport–dependent mechanism (Han et al., 2003).

### Dynein HC and IC abundances are reduced in Wdr92 mutant round spermatocytes

**Figure 4.**
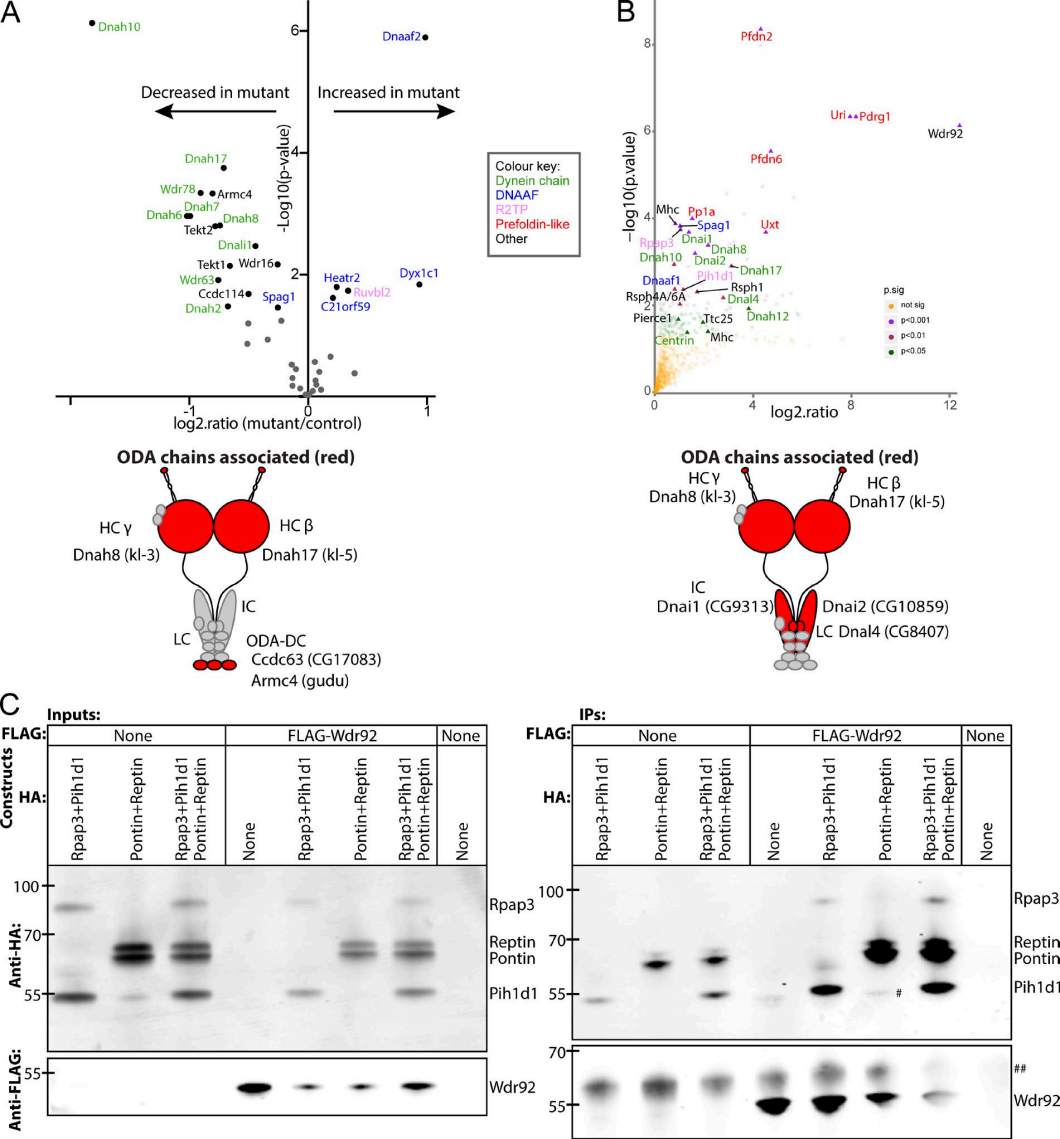
**Wdr92 associates with R2TP, dynein chains and DNAAFs. (A)** Quantitative proteomic profiling of whole spermatocyte extracts from control vs mutant testes during cytoplasmic axonemal dynein assembly represented as volcano plot of motility-associated proteins. Black dots are proteins significantly differentially present in wild-type (left) or mutant (right) spermatocytes (P < 0.05), with names color coded according to the key. Proteins are labeled according to their human orthology; see Table S1 for *Drosophila* gene names. Beneath is a schematic of the ODA complex with chains that are reduced shaded red. **(B)** Volcano plot of proteins associated with Wdr92-mVenus from AP-MS analysis. Significant proteins of interest are labeled (P < 0.05). Beneath is a schematic of the ODA complex chains associated with Wdr92 (red). **(C)** Wdr92 associates with the R2TP complex by coIP. S2 cells were transfected with plasmids encoding FLAG-tagged and HA-tagged *Drosophila* proteins and anti-FLAG was used in immunoprecipitation from cell extracts. Western blots of whole extracts (inputs) or of immunoprecipitates (IPs) were probed with anti-HA and anti-FLAG. The last lane is from mock-transfected S2 cells. # indicates a presumed degradation/truncation band for Pontin/Reptin; ## indicates a common nonspecific band.

To pinpoint when in cytoplasmic dynein assembly WDR92 function is required, we performed unbiased label-free mass spectrometry (MS) quantification of proteins in control versus *Wdr92^−/−^* mutants. We used extracts from 48-h pupal testes, a stage before sperm flagellum formation (Gärtner et al., 2014), to detect changes primarily reflecting Wdr92’s cytoplasmic role in dynein assembly/transport rather than secondary proteostatic effects of failure to populate flagella with motors. Interrogation of the MS data with a candidate list of 88 motility-associated proteins revealed that 40 could be detected in spermatocytes, and 20 of these appeared altered in abundance in mutant spermatocytes (P < 0.05; Fig. 4 A and Tables S1 and S2). Strikingly, these include a reduction in abundance of six dynein HCs representing all forms of ODA/IDA (ODA β and γ chains, IDA heterodimeric and monomeric forms) and several IDA ICs. Reduction is consistent with destabilization caused by failure in assembly of HC/IC complexes (Mitchison et al., 2012). The phenotype is notably different from mutants of several known DNAAFs, including *DNAAF2*, *DNAAF3*, and *ZMYND10*, in which HCs are reduced but ICs accumulate (Omran et al., 2008; Mitchison et al., 2012; Diggle et al., 2014).

Interestingly, several DNAAF orthologues were increased in the *Wdr92* mutant. Dnaaf2/Ktu (Nop17l in *Drosophila*) and Dyx1c1/Dnaaf4 (CG14921), which are proposed to form an R2TP-like cochaperone complex for an early HC assembly step, are both strongly increased. C21orf59/Kurly (CG18675) and Heatr2 (CG31320) are also increased. Surprisingly, there are reductions in homologues of dynein motor docking proteins: Ccdc63 (CG17083) and Armc4 (Gudu; ODA docking complex proteins), and two Tektins, which are implicated in IDA docking in *Chlamydomonas reinhardtii* and mouse (Tanaka et al., 2004; Yanagisawa and Kamiya, 2004).

### Wdr92 protein interacts with R2TP, prefoldin-like complex, and dynein chains

To obtain insight into the link to dynein motors, we determined the Wdr92 interactome in fly testes. Using GFP-trap affinity purification (AP), we immunoprecipitated Wdr92 from testes of *Wdr92-mVenus* adult males. As a negative control to exclude mVenus-interacting proteins, we performed AP-MS on extracts from testes overexpressing a ubiquitous, cytoplasmic Gap43-mVenus fusion protein. Proteins showing significant association with Wdr92 included several categories of interest for motor assembly, including known DNAAFs and PCD-causative genes, and dynein chains (Fig. 4 B and Table 1). First, AP-MS confirmed Wdr92 association with predicted R2TP and prefoldin-like complexes, reflecting previous proteomic analyses of human WDR92 interactors in cultured cells (Sardiu et al., 2008; Choi et al., 2011; Cloutier et al., 2017; Fig. 8).

**Table 1.** Protein interactors of Wdr92

***Drosophila* gene**	**Protein accession number**	**Human protein**	**Notes**	**Fold enrichment** ^a^	**Human interaction reported** ^b^
**R2TP/prefoldin-like complex**					
*spaghetti*	Q9V3E9	RPAP3	R2TP	2.06 (0.0002)	+
*CG5792*	Q9VK57	PIH1D1	R2TP	2.22 (0.0044)	+
*uri*	Q9W148	URI	PFDL	246.45 (4.51 × 10^−7^)	+
*l(3)01239*	M9PF40	PFDN2	PFDL	19.81 (4.4 × 10^−9^)	+
*CG7770*	M9PD09	PFDN6	PFDL	26.39 (2.8 × 10^−6^)	+
*l(2)35Cc*	Q9VJP9	UXT	PFDL	22.85 (0.0002)	+
*CG15863*	Q4QQ01	PDRG1	PFDL	291.12 (4.54 × 10^−7^)	+
*pp1-13C*	Q05547	PP1α	Uri interactor	2.87 (0.0001)	+
**DNAAFs**					
*dtr*	Q8INT5	DNAAF1	PCD	1.75 (0.0042)	−
*CG18472*	Q9VBA1	SPAG1	PCD	2.04 (0.0002)	−
**Axonemal dynein chains**					
*kl-5*	Q5LJN5	DNAH17	HC OADβ, PCD	8.66 (0.0012)	−
*kl-3*	A8Y5B7	DNAH8	HC OADγ, PCD	4.49 (0.0004)	−
*Dhc62B*	Q7KVA7	DNAH12	HC IAD, monomeric	14.12 (0.0119)	−
*Dhc98D*	E1JJ04	DNAH10	HC IAD, dimeric	1.72 (0.0011)	−
*CG9313*	Q8MSJ9	DNAI1	IC ODA, PCD	2.61 (0.0002)	−
*CG1085*9	Q9VJY4	DNAI2	IC ODA, PCD	3.11 (0.0006)	−
*CG3180*2	Q8T415	Centrin	IC IDA, monomeric	2.49 (0.0426)	−
*CG8407*	A1Z8T9	DNAL4	LC ODA	6.87 (0.0067)	−
**Other**					
*CG3121*	Q9W1D3	RSPH4A/6A	Radial spoke, PCD	2.03 (0.0094)	−
*CG5458*	Q9VK29	RSPH1	Radial spoke, PCD	3.29 (0.0049)	−
*Zipper*	Q59E58	MYH9/10	Myosin HC	4.48 (0.0403)	+
*CG34107*	Q0KI89	PIERCE1	LR asymmetry (mouse)	1.94 (0.0211)	−
*CG15128*	A1ZBP5	TTC25	Dynein docking, PCD	3.88 (0.0248)	−

LC, light chain; PFDL, prefoldin-like complex.

a Fold difference Wdr92 AP versus control AP (p-value).

b Homologous interaction reported for human WDR92 in cultured nonmotile ciliated cells (Choi et al., 2011).

Critically, AP-MS provides the first evidence that Wdr92 associates with ciliary motility proteins. This includes dynein chains, which may be clients for Wdr92/R2TP cochaperone activity, and DNAAFs homologues, which may be functional partners. Dynein chains include HC and ICs of all forms of ODA and IDA. A single light chain is associated with Wdr92: the homologue of DNAL4. This is a component of the ODA, but it is also known that mutation of the homologous subunit in *Chlamydomonas* (LC10) causes defects in ODA assembly (Tanner et al., 2008). Surprisingly, two radial spoke proteins are present among the interactors (RSPH4A/6A and RSPH1). The protein interaction data provide evidence supporting a role for Wdr92 in bringing dynein client proteins to the R2TP cochaperone.

### R2TP complex is required for dynein arm assembly; prefoldin-like complex is not

R2TP comprises two parts: a heterodimer of Rpap3 and Pih1d1 (Spaghetti and CG5792 in *Drosophila* (Benbahouche et al., 2014), and a heteromultimer of helicases RUVBL1/Pontin and RUVBL2/Reptin, although these latter additionally play many non-R2TP roles (Sardiu et al., 2008; Choi et al., 2011; Kakihara and Houry, 2012; Nano and Houry, 2013; Cloutier et al., 2017; Fig. 8). In coimmunoprecipitation (coIP) experiments of *Drosophila* S2 cells transfected with constructs to overexpress tagged proteins, we found that *Drosophila* Wdr92 physically interacts with Rpap3/Pih1d1, confirming the AP-MS results above and consistent with the known human interactions (Sardiu et al., 2008; Choi et al., 2011; Cloutier et al., 2017; Fig. 4 C). It is likely that Wdr92 binds Rpap3/Spaghetti directly via the latter’s C-terminal RPAP_3C domain; however, in S2 cells, Wdr92 associated with Pih1d1 as well as Rpap3, probably through bridging via endogenous Rpap3 (Fig. S1). Although we did not detect Reptin or Pontin as Wdr92 interactors by AP-MS, we could confirm interaction with Pontin/Reptin in S2 cells (Fig. 4 C). These interactions may similarly be facilitated by endogenous Rpap3 protein.

**Figure 5.**
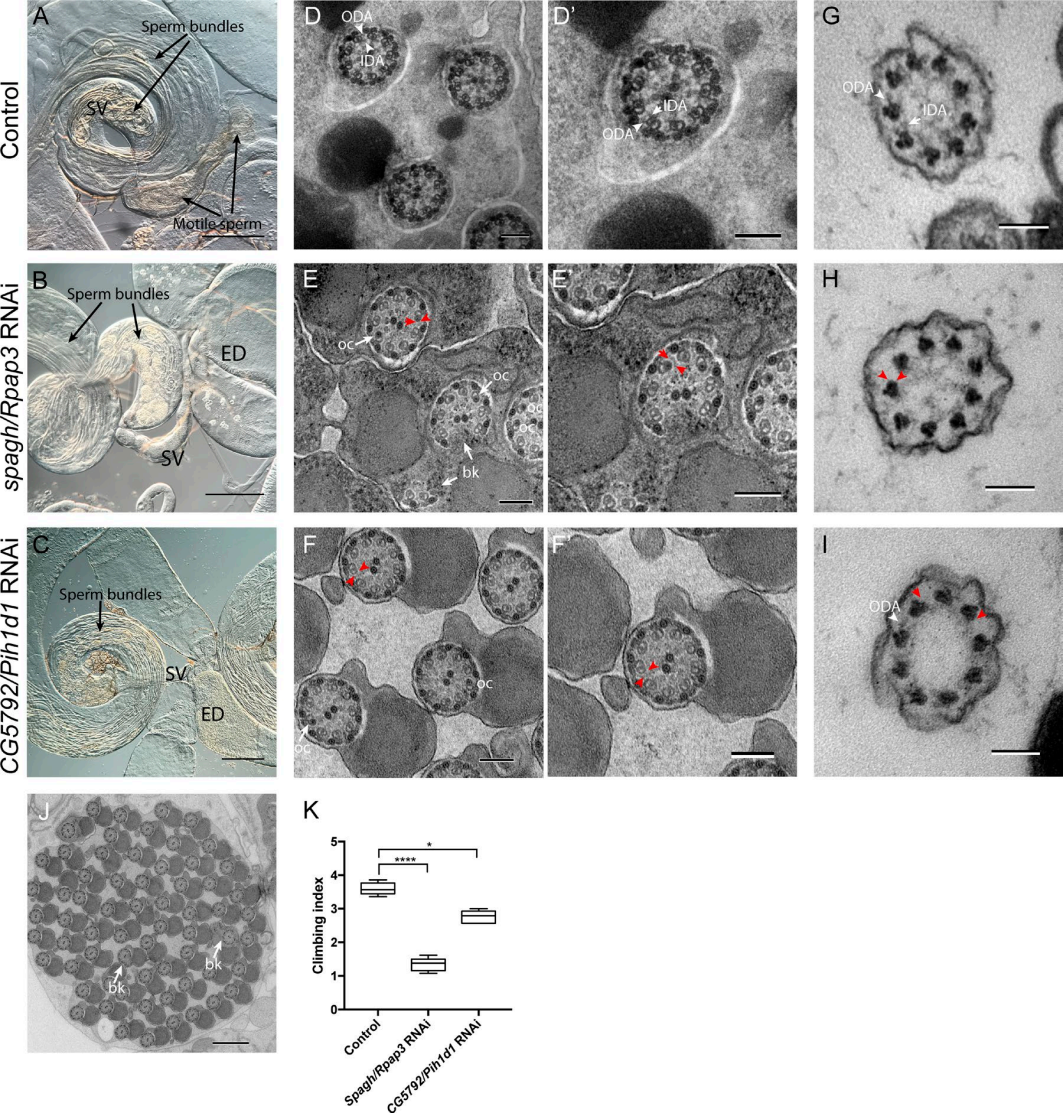
**R2TP complex is required for dynein arms. (A–C)** Adult male reproductive organs. **(A)** Control showing sperm bundles and motile sperm in SV. **(B and C)** Testes-specific RNAi depletion of *spaghetti/Rpap3* (B) and *CG5792/Pih1d1* (C), showing sperm bundles but absence of motile sperm in SVs or ED. **(D–F′)** TEM of adult testes, transverse sections. **(D and D')** Control flagella, showing presence of ODA/IDA (white arrowheads). **(E and F)** RNAi depletion of *spaghetti/Rpap3* (E and E') and *CG5792/Pih1d1* (F and F') showing largely normal axonemal structures, but with loss of ODA/IDA (red arrowheads show examples where expected), occluded A microtubules (oc), and axonemal breakages (bk). **(G and I)** Transmission electron micrographs of antennal Ch neuron cilia, transverse sections. **(G)** Control showing ODA/IDA. **(H and I)** RNAi knockdown of *spaghetti/Rpap3* (H) and *CG5792/Pih1d1* (I), showing grossly normal cilia with complete (H) or partial (I) loss of ODA/IDA (red arrowheads). **(J)** Section through entire sperm bundle from *CG5792/Pih1d1* depleted male, showing grossly normal flagella of 65 sperm (expected number 64). Some axonemal breakages are observed (bk). Bars: (A–I) 100 nm; (J) 500 nm. **(K)** *CG5792/Pih1d1* and *spaghetti/Rpap3* RNAi-depleted flies show defective response in climbing assay. Box plot (median and interquartile range) of adult climbing assay for RNAi lines crossed to *sca*Gal4 driver. Significance was determined by Kruskal-Wallis test, with Dunn’s test for multiple comparisons. Significance on plots is signified by asterisks: *, P ≤ 0.05; ****, P ≤ 0.0001.

Consistent with the complex’s multiple roles, *Drosophila* homologues of R2TP subunits are not strongly enriched in Ch neurons, and mutations are lethal (Table S3). To investigate a possible role for R2TP in dynein motor assembly and to overcome cell-vital functions, we performed tissue-specific RNAi depletion in spermatocytes for the R2TP-unique subunits, *spaghetti/Rpap3* and *CG5792/Pih1d1*. In each case, we observed complete male infertility (*n* = 10 males) with testes that appeared to contain largely normal sperm bundles but lacked motile sperm (*n* = 7 for *CG5792*, *n* = 11 for *spaghetti*; Fig. 5, A–C). TEM confirmed relatively normal sperm bundles (Fig. 5 J) and flagella (Fig. 5, D–F) but with strong loss of dynein arms as well as the occurrence of A tubule occlusions and axonemal fragmentation (Fig. 5, D–F). Depletion of either gene in developing sensory neurons also resulted in normal cilium appearance but with loss of dynein arms (Fig. 5, G–I), although this was incomplete for *CG5792* (*spaghetti*: 9/12 cilia showed loss of arms; *CG5792*: 7/20 cilia showed partial loss of arms). When proprioception was assayed in flies raised at 28.5°C (enhancing the efficiency of RNA depletion), a stronger behavioral defect was observed for both subunits (Fig. 5 K), suggesting that RNAi is inefficient for this line. These phenotypes suggest that a major role of R2TP in motile ciliated cells is to promote dynein arm assembly.

**Figure 6.**
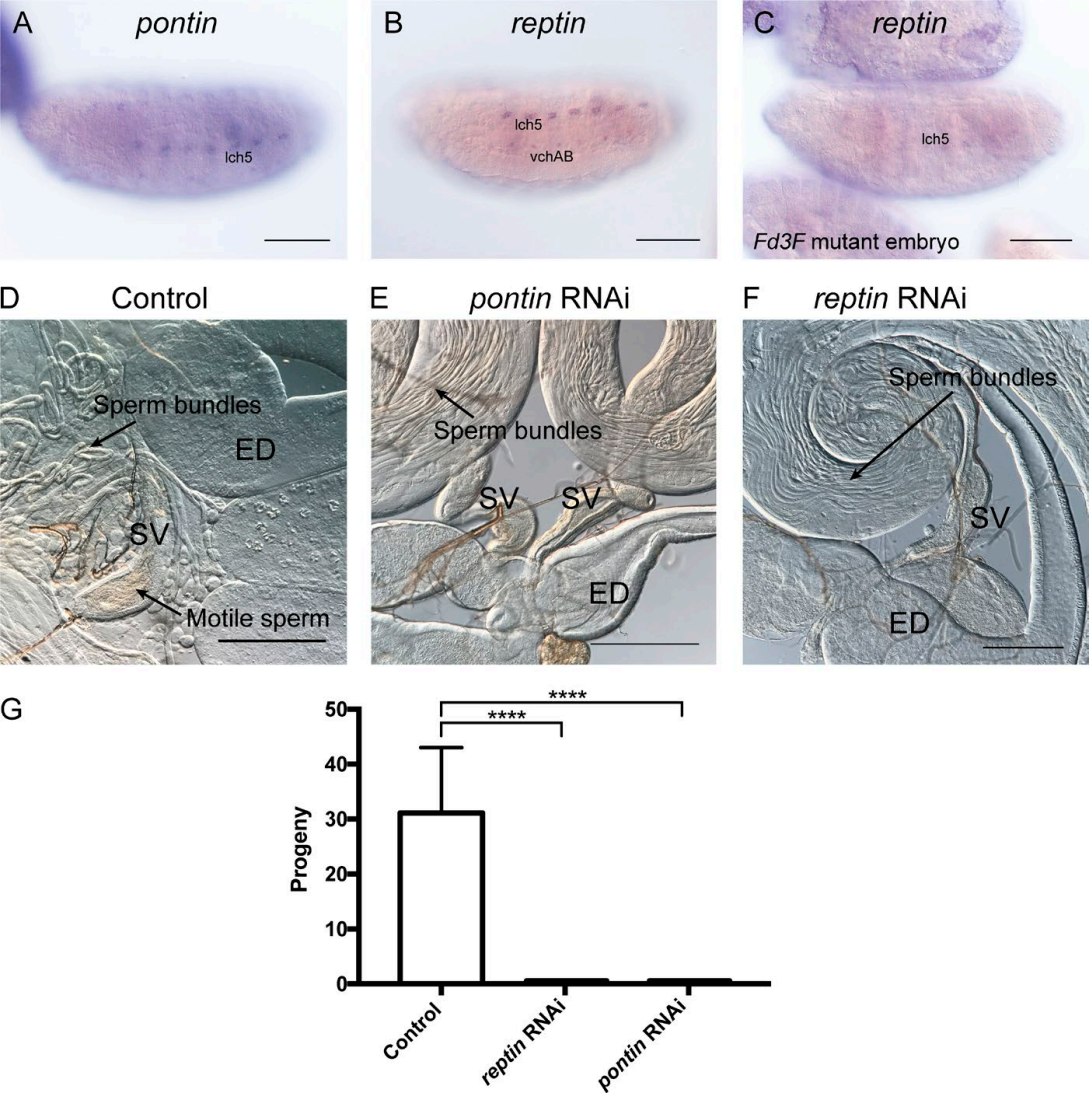
***pontin* and *reptin* are required for motile sperm. (A–C)** RNA in situ hybridization in stage 16 embryos. **(A and B)** *pontin* and *reptin* expression is strongest in Ch neurons. **(C)** For *reptin*, this expression is much reduced in embryo mutant for *Fd3F*, which encodes the transcription factor that regulates motile cilia genes. **(D–F)** Adult male reproductive organs. **(D)** Control showing sperm bundles in testis and motile sperm in SV. **(E and F)** RNAi depletion of *pontin* and *reptin* results in loss of motile sperm from SV even though normal sperm bundles are present. **(G)** RNAi depleted males are infertile: mean and SD of progeny per male, *n* = 10 males. Significance was determined by ordinary one-way ANOVA, with Dunnett’s test for multiple comparisons; significance on plot is signified by asterisks: ****, P ≤ 0.0001. Bars, 100 µm.

Despite their widespread functions, both *reptin* and *pontin* are enriched in Ch neurons, suggesting a stronger requirement than in other cells (Fig. 6, A and B), and the enriched expression of *reptin* in Ch neurons appears to be partially dependent on the motile cilia transcriptional regulator, Fd3F (Fig. 6 C). RNAi depletion of either *reptin* or *pontin* in *Drosophila* testes indeed resulted in infertility (Fig. 6 G) and specific absence of motile sperm (*n* = 10 flies) (Fig. 6, D and E), suggesting that their major role in sperm differentiation is concerned with dynein motors. We were unable to assess the function of these genes in Ch neurons, because embryonic depletion of either gene proved to be lethal.

The AP-MS data show that Wdr92 strongly associates with the five subunits of the prefoldin-like complex (Fig. 4 B), in agreement with interactions previously noted for *Drosophila* and human WDR92 (Sardiu et al., 2008; Glatter et al., 2011). The prefoldin-like complex shares two subunits (PFDN2 and PFDN6) with the canonical prefoldin complex, which is best known for assisting in the folding of actin and α- and β-tubulin through interaction with the Chaperonin-containing TCP-1 complex (Millán-Zambrano and Chávez, 2014). Three subunits are unique to the prefoldin-like complex (URI1, PDRG1, UXT), which we investigated here. Surprisingly, RNAi depletion of each of these subunits resulted in neither proprioceptive defects (when depleted in sensory neuron precursors) nor male infertility nor sperm immotility (when depleted in spermatocytes; *Uri*, 4 independent RNAi lines; *Uxt*, 3 lines; *Pdrg1/CG15863*, 1 line; for sperm motility, *n* = 10 males for each line). This suggests that despite its proteomic link with Wdr92/R2TP, the prefoldin-like complex is not involved in dynein arm assembly. Moreover, unlike Wdr92, there is no indication that expression of *Drosophila* prefoldin-like genes is enriched in ciliated cells, either in the Ch neuron transcriptome or in testis (Table S3).

### Wdr92 interacts with CG18472, an orthologue of DNAAF, Spag1

**Figure 7.**
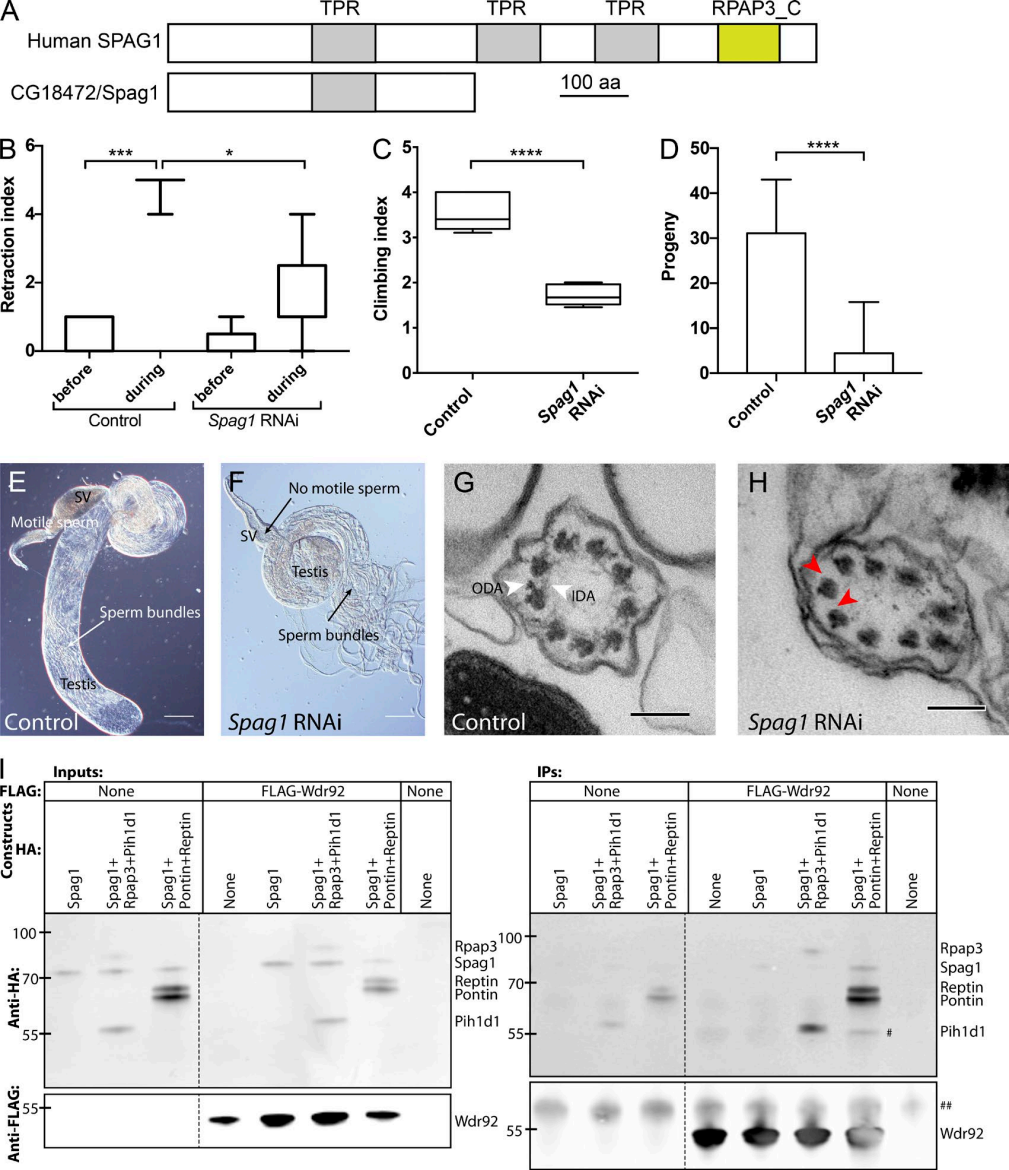
***CG18472/Spag1* is required for dynein assembly. (A)** Schematic comparison of human SPAG1 and *Drosophila* CG18472/Spag1 proteins. **(B)** *CG18472/Spag1*-depleted larvae are deaf in a larval hearing assay. Box plot (median and interquartile range) of RNAi line crossed to *sca*Gal4 driver, *n* = 9 batches of five larvae. **(C)** *Spag1*-depleted flies show poor proprioception in a climbing assay. Box plot (median and interquartile range) of RNAi line crossed to *sca*Gal4 driver, *n* = 5 batches of 15 flies. **(D)** *Spag1*-depleted males produce few progeny. Mean and SD of progeny per male, *n* = 10 males. Significance was determined by Kruskal-Wallis test, with Dunn’s test for multiple comparisons (B and C) or ordinary one-way ANOVA, with Dunnett’s test for multiple comparisons (D). Significance on plots is signified by asterisks: *, P ≤ 0.05; ***, P ≤ 0.001; ****, P ≤ 0.0001. **(E)** Control adult testis showing SV filled with motile sperm. **(F)** *Spag1*-depleted testis showing sperm bundles but lack of motile sperm in SV. Bars, 100 µm. **(G and H)** TEM of antennal Ch neuron cilium (transverse section), showing loss of dynein arms (ODA, IDA) after *Spag1* depletion (H) compared with control (G). Red arrowheads indicate examples of where ODA/IDA should be. Bars, 100 nm. **(I)** *Drosophila* Wdr92 and Spag1 associate by coIP. S2 cells were transfected with plasmids encoding FLAG-tagged and HA-tagged *Drosophila* proteins, and anti-FLAG was used in immunoprecipitation from cell extracts; Western blots of whole extracts (inputs) or immunoprecipitates (IPs) were probed with anti-HA and anti-FLAG. The last lane is from mock transfected S2 cells. # indicates a presumed degradation/truncation band for Pontin/Reptin; ## indicates a common nonspecific band.

By AP-MS, the orthologues of two DNAAFs associate with Wdr92. This includes CG18472, the closest *Drosophila* homologue of human SPAG1 (Knowles et al., 2013; 49% similarity). Like RPAP3, human SPAG1 has an N-terminal TPR that may associate with HSP90 and a C-terminal potential WDR92-binding RPAP3_C domain. Indeed, recent proteomic analysis supports an association between human WDR92 and SPAG1 (Cloutier et al., 2017). However, the C-terminal domain is not conserved in *Drosophila* CG18472 (Fig. 7 A; nor in other insect homologues). Despite this difference, we obtained several lines of evidence that suggest the CG18472 protein conserves the dynein assembly function of SPAG1. First, *CG18472/Spag1* mRNA is highly expressed in the differentiating Ch neuron transcriptome (17.3-fold enriched; Cachero et al., 2011) and in testis. Flies in which *CG18472/Spag1* was depleted in Ch neurons had defective larval hearing (Fig. 7 B) and adult proprioception (Fig. 7 C), whereas depletion in testes caused complete male sterility (Fig. 7 D) with mature but immotile sperm (*n* = 8 flies; Fig. 7, E and F). In addition, by TEM, Ch neuron cilia, and sperm flagella lacked ODA/IDA (22/25 cilia showed complete loss of arms; Fig. 7, G and H). We conclude that CG18472/Spag1 likely conserves the dynein assembly function of human SPAG1.

To explore the Wdr92-Spag1 association, we conducted AP-MS on Spag1 from testes of adult males with a *Spag1-mVenus* fusion gene. Strikingly, Wdr92 showed significant association (ratio vs. control: 11.1; P = 0.003711). The only dynein-related protein showing significant association with Spag1 was Dhc98D (Dnah10), an inner arm dynein HC that was also associated with Wdr92 (ratio vs. control: 2.4; P = 0.021265) and strongly reduced in *Wdr92* mutant spermatocytes (Fig. 4 A). Interestingly, in S2 cell coIP experiments, Spag1 association with Wdr92 was facilitated by the presence of overexpressed Pontin and Reptin (Fig. 7 I). We suggest that Wdr92-Spag1 form part of a conserved cochaperone complex. It is notable that Spag1 protein abundance is decreased in *Wdr92^−/−^* testes (Fig. 4 A), consistent with codependence of the two proteins in a complex.

## Discussion

**Figure 8.**
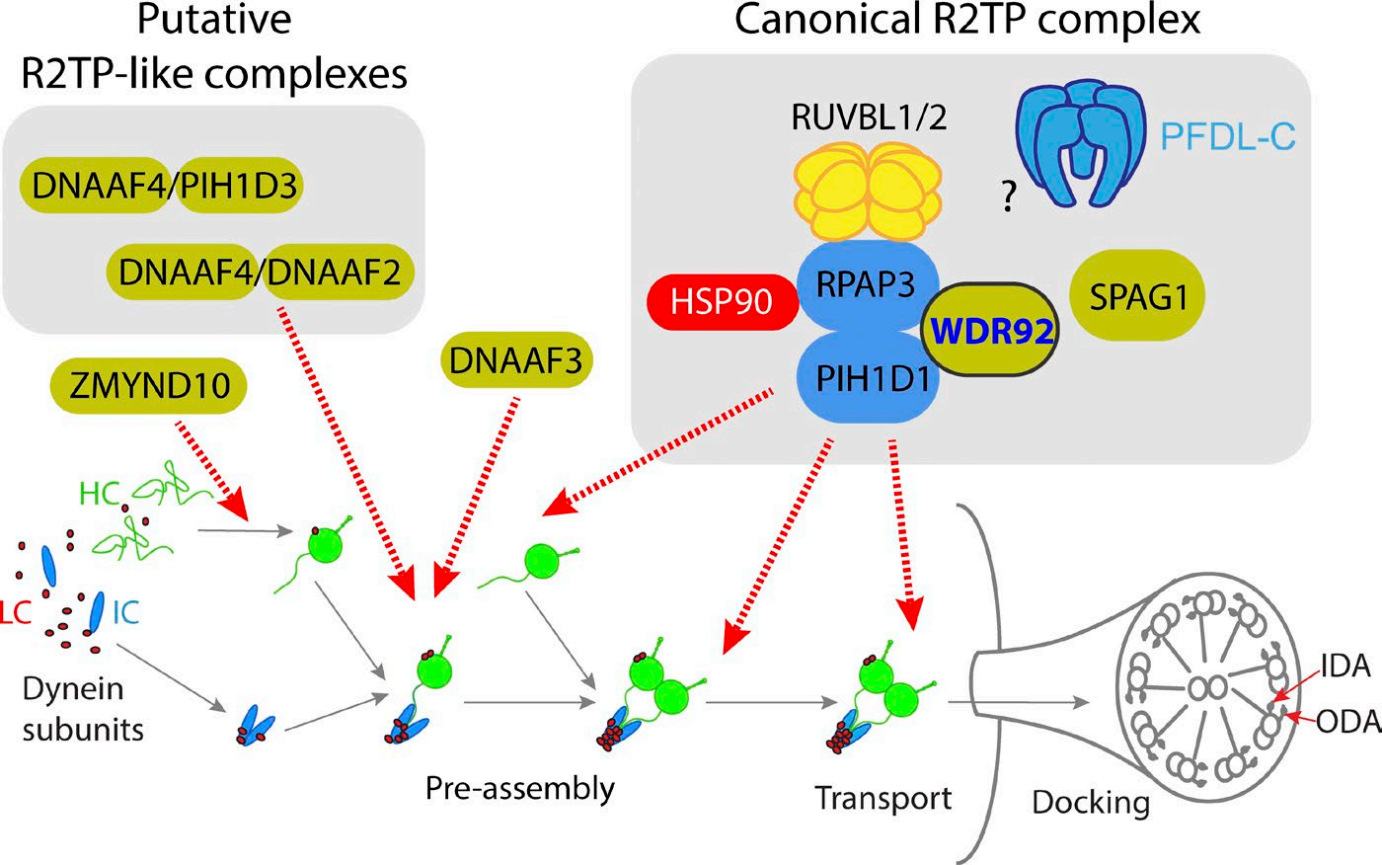
**Summary of *Wdr92* interactions and proposed functions.** Schematic summary of the putative dynein preassembly pathway, with proposed locations of chaperone action of several DNAAFs indicated (orange). Some DNAAFs have been proposed to form R2TP-like complexes, although this is not proven. In contrast, Wdr92/Spag1 are hypothesized to act at a late stage of complex assembly and/or handover to trafficking pathways. This is mediated via canonical R2TP complex, but the prefoldin-like complex (PFDL-C) appears not to be involved. HSP90 is known to bind to R2TP, but was not detected in this study.

Although WDR92’s proteomic association with the HSP90 cochaperone, R2TP, has been known for some time, very little was known of the function of WDR92 or the significance of this association in vivo (Sardiu et al., 2008; Boulon et al., 2010; Choi et al., 2011; Glatter et al., 2011). We found that *Drosophila* Wdr92 protein is specifically expressed in motile ciliated cells before their terminal differentiation and is confined to the cytoplasm, where dynein preassembly takes place. Null mutants are viable, with specific loss of ODA and IDA from otherwise normal cilia/flagella. In these features, *Wdr92* resembles fly homologues of known DNAAFs (Kavlie et al., 2010; Moore et al., 2013; Diggle et al., 2014), suggesting that *Wdr92* in *Drosophila* is a dedicated DNAAF (Fig. 8).

R2TP is the most complex HSP90 cochaperone, with its PIH1D1 subunit thought to impart specificity for a variety of clients including TOR, snoRNP, and RNA polymerase II (Eickhoff and Costa, 2017). We provide the first direct evidence that dynein chains are also R2TP clients. Previous studies of R2TP could not reveal interactions with dynein chain clients or DNAAFs because proteomic analyses were performed on cell lines lacking motile cilia. Mutations of *spaghetti* (which encodes the RPAP3 subunit of *Drosophila* R2TP) are lethal: *spaghetti* null mutant embryos survive owing to maternal contribution, but the larvae then die with atrophy of most organs, which can be explained by its multifunctional cochaperone role (Benbahouche et al., 2014). In contrast, Wdr92 function appears confined to dynein assembly, suggesting that it is an adaptor specifically required to regulate the dynein assembly function of R2TP. We propose that the DNAAF, SPAG1, functions in close association with WDR92, although it remains to be determined whether WDR92-SPAG1 work together with R2TP in a single complex or WDR92-SPAG1 form an independent cochaperone complex.

Based on their possession of TPR and PIH domains, several DNAAFs have been speculated to form cochaperone complexes akin to R2TP (known as R2TP-like complexes), including DNAAF4-DNAAF2 and DNAAF4-PIH1D3 (Tarkar et al., 2013; Pal et al., 2014; Vaughan, 2014; Olcese et al., 2017; Paff et al., 2017). Moreover, studies of RUVBL1/2 requirement for dynein assembly are interpreted as evidence for their involvement in these hypothetical cilium-dedicated R2TP-like complexes (Zhao et al., 2013; Li et al., 2017). It is notable that we found no evidence of physical interaction between Wdr92 and these DNAAFs by AP-MS (nor by direct coIP experiments in S2 cells; unpublished data), suggesting that Wdr92/R2TP is a distinct entity in the dynein assembly pathway. Although evidence for R2TP-like complexes currently remains inconclusive, our study establishes that canonical R2TP is required for dynein assembly. It is possible, therefore, that previous studies of RUVBL1/2 requirement in dynein assembly indicate their involvement with canonical R2TP. Interestingly, DNAAF4 and DNAAF2 proteins were both increased in abundance in *Wdr92* mutant spermatocytes, perhaps in attempted compensation for loss of Wdr92-mediated cochaperone activity.

In mutant analyses of several DNAAFs (notably *DNAAF2* and *DNAAF3*), reductions in dynein HC abundances have been interpreted as representing instability and clearance of partly assembled motor complexes caused by incorrect HC chaperoning, whereas increase in IC abundances represents accumulation of an IC-based subcomplex before its assembly with HCs (Omran et al., 2008; Mitchison et al., 2012; Moore et al., 2013). *Wdr92* mutants are unique in exhibiting reductions in HCs but no accumulation of ICs (indeed, many ICs are reduced), and Wdr92 physically associates with both HCs and ICs. This suggests that it defines a distinct step in dynein assembly, perhaps at a late stage in the process. Several surprising associations with proteins involved in aspects of axonemal motor docking may suggest also a role in late stage complex handover for trafficking to the cilium. This includes physical interaction with radial spoke proteins that interact with docked IDAs, as well as reduced abundance in the *Wdr92* mutant of outer arm docking complex subunits (ODA-DC) and tektins, which are implicated in inner arm docking in *Chlamydomonas* and mouse (Tanaka et al., 2004; Yanagisawa and Kamiya, 2004). Because tektins are also thought to line the lumen of the microtubule doublets, it is possible that disruption in tektin assembly/trafficking underlies the occluded A tubule phenotype seen in mutants of *Wdr92*, R2TP, and other DNAAF homologues in *Drosophila*.

Proteomic analyses (including the present study) consistently demonstrate coassociation of WDR92 with the prefoldin-like proteins, and on this basis, Wdr92 is often considered to be part of a prefoldin-like complex (Millán-Zambrano and Chávez, 2014). It is therefore curious that we found no functional evidence for involvement of the prefoldin-like complex in dynein preassembly, in contrast to R2TP. In fact, there is no functional evidence that human WDR92 is required for prefoldin-like functions. None of the *Drosophila* subunits of the prefoldin-like complex are enriched in motile ciliated cells, and mutations in their genes are lethal, suggesting a role in multiple pathways (*uri*, *uxt/l(2)35Cc*, and *Pdrg1/l(3)01239* are all lethal mutations). Perhaps consistent with a lack of involvement in dynein assembly, previously it was shown that *Drosophila uri* mutants with a partially rescued soma function exhibit sperm defects but still produce motile sperm (Kirchner et al., 2008). In addition, only *Wdr92* is phylogenetically associated with organisms with motile cilia (Baron et al., 2007). For these reasons, we suggest that Wdr92, at least in *Drosophila*, is not a core component of a prefoldin-like complex. Moreover, the existence of a prefoldin-like complex and its function as a chaperone are unclear. The proposal of a prefoldin-like complex as a chaperone derives largely by analogy with the canonical prefoldin chaperone complex (Millán-Zambrano and Chávez, 2014), with which it shares two subunits (PFDN2 and PFDN6). Apart from its proteomic association with protein folding/stability processes (e.g., RNA polymerase assembly, PIKK stabilization), there is so far little direct functional evidence for involvement (although the URI homologue, *bud27*, participates in RNA polymerase II assembly in yeast; Mirón-García et al., 2013). In contrast, functional evidence suggests several prefoldin-like subunits act independently in several processes, such as transcriptional elongation (Millán-Zambrano and Chávez, 2014; Mirón-García et al., 2014).

In human and mouse, WDR92 appears to be widely expressed and not restricted to tissues with motile cilia. Indeed, in mammals, the proteomic association of WDR92 with R2TP/prefoldin-like extends to cells without motile cilia (Mita et al., 2013). It is possible that mammalian WDR92 has nonmotile cilia roles, with or without a prefoldin-like complex. Nevertheless, WDR92 (and RPAP3) appear with highest abundance in mouse testis just before flagellogenesis (http://fantom.gsc.riken.jp/5/sstar/EntrezGene:103784). Combined with the comparative genomic link between Wdr92 and ciliary motility, it is plausible to propose that dynein assembly is a conserved major function of WDR92 in other organisms including humans, even if not the sole function.

## Materials and methods

### Fly stocks

Flies, unless stated otherwise, were maintained on standard cornmeal agar media at 25°C. The stocks *UAS-Dcr-2; scaGal4* and the Cas9 injection line *vasa::Cas9* (Bl#51323) were obtained from the Bloomington Stock Center (Indiana University, Bloomington, IN). RNAi lines (Table S4) were obtained either from Bloomington (TRiP lines) or from the Vienna *Drosophila* Resource Centre. *BamGal4*-VP16 stock was a gift from H. White-Cooper (Cardiff University, Cardiff, Wales, UK). Control flies were the appropriate RNAi line parent stock (Table S4) or Oregon-R. Other stocks used were *Fd3F^1^* (Newton et al., 2012) and Dnali1-mVenus (CG6971-mVenus; Diggle et al., 2014).

### Behavioral assays

The adult climbing assay and larval hearing assay were performed as previously described (Diggle et al., 2014). A 20-cm tube was divided into four sections and flies scored according to the section climbed in 15 s. Adults were tested in replicate batches of 10–15 with the mean representing the climbing index. *n* = 5 batches unless otherwise stated. Larvae were tested in replicate batches of five for contraction response before and during a tone of 1000 Hz, and the number of responding larvae were aggregated as the response score for that replicate. *n* = 9 replicates unless otherwise stated. Data are plotted as box plots with whiskers representing minimum and maximum values.

### Male fertility assay

RNAi flies were crossed to *BamGal4-VP16*. From the progeny, 2- to 5-d-old males (*n* = 10) were crossed to Oregon-R females and allowed to deposit eggs for 2 d. Flies were then transferred to new vials to lay eggs for 2 d. Progeny from the latter were counted. Data are plotted as bars with mean and SD.

### RNA in situ hybridization

DIG-labeled UTP (DIG RNA Labeling Mix; 11277073910; Roche) antisense RNA probes were generated using T7 RNA polymerase (10881767001; Roche) from a PCR product (400–500 bp) containing the T7 RNA polymerase promoter at its 3′ end. Embryos or testes(Morris et al., 2009) were fixed in 4% formaldehyde and prehybridized (50% deionized formamide [vol/vol], 5× SSC, 10 mg/ml tRNA, 50 mg/ml heparin, and 0.1% Triton X-100, pH 6.5). Hybridization was performed at 70°C overnight. Tissues were washed in PBS with 0.1% Igepal CA-630 (Sigma-Aldrich). After incubation with anti–digoxigenin-AP antibody (1:2,000; 1093274910; Roche), the staining pattern was visualized by incubating in reaction buffer (100 mM Tris, pH 9.5, and 100 mM NaCl with the NBT/BCIP color reagent; 11681451001; Roche). After washing, tissues were mounted in 80% glycerol.

### Immunofluorescence

For tissue stainings, tissues were fixed in 4% formaldehyde for 10–20 min (Newton et al., 2012), washed in phosphate buffered saline with 0.3% Triton X-100 (PBT), then blocked in PBT with 2% bovine serum albumen for 2 h. Tissues were then incubated with primary antibody in PBT overnight, washed in PBT, and incubated with secondary antibody for 2 h. After washing, tissues were mounted in VectorShield (Vector Laboratories). Antibodies used were rabbit anti-GFP (1:500; A-11122; Thermo Fisher Scientific), mouse anti-Futsch/mAb-22C10 (1:200; Developmental Hybridoma Bank Iowa), rabbit anti-HRP (1:500; Jackson Laboratories), and mouse anti-NompC (1:200; a gift from X. Liang, Yale University, New haven, CT; Liang et al., 2011). Secondary antibodies (Thermo Fisher Scientific), Alexa Fluor 488 goat anti–rabbit (A-11008), Alexa Fluor goat anti–mouse 488 (A-11001), Alexa Fluor 568 goat anti–rabbit (A-11036), and Alexa Fluor 568 goat anti–Mouse (A-11019) were used at a concentration of 1:500. TO-PRO3 (Thermo Fisher Scientific) was used at 1:1,000.

### Fluorescence and brightfield microscopy

Fluorescence images were acquired using a Zeiss LSM510 confocal system with Axioskop2 or Axiovert microscope, using Zeiss LSM510 software with the following objectives: Plan Neofluar 10×/0.3, Plan Neofluar 20×/0.5, and Plan Apochromat 63×/1.4 Oil. Brightfield images were acquired using a Olympus Provis AX-70 microscope with an Olympus DP50 camera, with the following objectives: UPlanApo 10×/0.4 and UPlanApo 20×/0.7. In all cases, images were processed for gamma adjustment using FIJI software.

### TEM

Whole adult heads were removed and rinsed in 0.5% Triton X-100. The proboscis was removed to facilitate infiltration of the fixative, and the heads were then fixed in 2.5% glutaraldehyde and 2% paraformaldehyde in 0.1 M phosphate buffer, pH 7.4, overnight at 4°C. Heads were then washed in 0.1 M phosphate buffer, pH 7.4, postfixed with OsO_4_, dehydrated in an ethanol series, and embedded in Polybed812. Ultrathin (75 nm) sections of the antennae were then stained with aqueous uranyl-acetate and lead citrate and examined with a Hitachi 7000 electron microscope.

### mVenus fusion gene construction

A *Wdr92-mVenus* fusion gene was designed as follows: the *Wdr92* gene was PCR-amplified from genomic DNA using primers designed to include the upstream region containing predicted Rfx and Fd3F binding sites to allow expression from its own promoter. The PCR fragment was cloned into pDONR221 via a BP Gateway reaction (Life Technologies). A Gateway LR clonase reaction then transferred this insert into the pBID-UASC-GV destination vector (Wang et al., 2012) to generate vector pBID-UASC-CG14353::mVenus. Transformant fly lines were generated by microinjection into syncytial blastoderm embryos of the attP40 landing site line. A similar strategy was used for *Spag1-mVenus* generation. Primers are listed in Table S4.

### AP and identification of proteins from testes

Three replicates of 150 pairs of testes were dissected from *Wdr92-mVenus* or *Spag1-mVenus* adults, the wild-type control line *w^1118^*, and the mVenus control *UAS-GAP43-mVenus* crossed to *BamGal4*. The testes were homogenized on ice for 2 min in lysis buffer (50 mM Tris-HCl, pH 7.5, 100 mM NaCl, 10% glycerol, 5 mM EDTA, 0.15% Triton X-100, and 0.5% sodium deoxycholate) in the presence of Complete Protease Inhibitor (Roche). Samples were rotated for 30 min at 4°C, before spinning for 10 min at 14,000 rpm at 4°C. The lysate supernatant was transferred to clearing beads, Sepharose beads IgG Fastflow (GE), and incubated for 30 min at 4°C before adding them to GFP-Trap_A beads (Chromotek) and rotating them for a further 3 h at 4°C. Subsequently, the beads were washed twice in lysis buffer, twice in lysis buffer containing 0.2% sodium deoxycholate, and twice more in lysis buffer. MS was performed on these samples as previously described (Turriziani et al., 2014). In brief, the samples were digested on-beads, and analyzed by liquid chromatography tandem MS on a Q-Exactive Plus (Thermo Fisher Scientific). Raw data were searched with MaxQuant against the *Drosophila* reference proteome with M(ox) and N-terminal acetylation as variable modifications. Protein quantification was done by MaxLFQ (Wiśniewski and Mann, 2012). For imputation and statistical analysis, the Perseus software suite was used.

### Protein expression analysis of testis by MS

For protein abundance experiments, 48-h pupal testes were dissected from 15 pupae per replicate for *Wdr92^X2^* mutant or control pupae. Samples were collected in lysis buffer containing 2 M urea, 25 mM dithiothreitol, and 125 mM Tris-HCL, pH 7.5. Lysates were then incubated for 30 min at 50°C. Reduced cysteine residues were alkylated by adding iodoacetamide solution to a final concentration of 50 mM and incubated 30 min at room temperature, in the dark. Proteins were digested with by adding 0.1 µg Trypsin (Promega) per sample for 16 h at 37°C. Trypsin activity was inhibited by acidification of samples to a concentration of 1% trifluoroacetic acid. Digests were clarified by centrifugation (20,000 *g*, 5 min), samples were desalted on a C18 Stage tip, and eluates were analyzed by HPLC coupled to a Q-Exactive Plus mass spectrometer as described above but with an extended gradient of 120 min. Peptides and proteins were identified and quantified with the MaxQuant software package (1.5.7.4), and label-free quantification was performed by MaxLFQ (Wiśniewski and Mann, 2012). The search included variable modifications for oxidation of methionine, protein N-terminal acetylation, and carbamidomethylation as fixed modification. The false discovery rate, determined by searching a reverse database, was set at 0.01 for both peptides and proteins. All bioinformatic analyses were performed with the Perseus software. Intensity values were log-normalized, and 0-values were imputed by a normal distribution 1.8 π down of the mean and with a width of 0.2 π. The MS proteomics data have been deposited to the ProteomeXchange Consortium with the dataset identifier PXD006935.

### Transfection and coIP of S2 cells

cDNAs were synthesized from antennal or testis mRNA (a gift from F. Newton, University of Edinburgh, Edinburgh, Scotland, UK) and cloned into the C-terminal site of plasmids pAWH (3x HA epitopes) and pAWF (3x FLAG epitopes) of the *Drosophila* Gateway Vector collection (Carnegie Institution for Science). Primers for synthesis are in Table S4. Transfection into S2 cells was performed using X-TREME GENE HP DNA transfection reagent (Roche). The cells were harvested after 48–72 h, and coIP was performed according to the FLAG Immunoprecipitation kit (Sigma-Aldrich). After Western blotting, the polyvinylidene fluoride membrane was incubated with mouse anti-FLAG M2 antibodies (1:1,000; F1804; Sigma-Aldrich) and rabbit anti-HA (1:4,000; ab9110; Abcam) antibodies. Secondary antibodies were supplied by Li-COR (IR Dye 680RD and IR Dye 800CW), and protein detection was performed on a Li-COR Odyssey scanner using ImageStudio v5.2 software.

### Statistical analysis

Data were plotted and analyzed using Prism 7 (Graphpad Software, Inc.). For climbing and hearing assay data, significance was determined by the Kruskal-Wallis test, with Dunn’s test for multiple comparisons. For male fertility data, significance was determined by ordinary one-way ANOVA, with Dunnett’s test for multiple comparisons; data distribution was assumed to be normal, but this was not formally tested. Significance on plots is signified by asterisks: *, P ≤ 0.05; **, P ≤ 0.01; ***, P ≤ 0.001; ****, P ≤ 0.0001.

### *CG14353* CRISPR mutant generation

A *CG14353* CRISPR/Cas9 mutant (*Wdr92^x2^*) was constructed by mini-*white* gene substitution according to Vieillard et al. (2016) using the following primers: RNA guide oligonucleotide 1 sense 5′-CTTCGCTTATTGAGCACCTCCACG-3′, RNA guide oligonucleotide 1 antisense 5′-AAACCGTGGAGGTGCTCAATAAGC-3′, RNA guide oligonucleotide 2 sense 5′-CTTCGGCAGTACGAGTATCCCGAC-3′, and RNA guide oligonucleotide 2 antisense 5′-AAACGTCGGGATACTCGTACTGCC-3′. The upstream homology arm primers contained an EcoRI and a *Asp*718I site (underlined), respectively: 5′-GGGGAATTCTTTTATTCGCTTCGTTGTGG-3′ and 5′-GATGGTACCGGAGGTGCTCAATAAGCTG-3′. The downstream homology arm primers contained a SpeI and a XhoI site, respectively: 5′-CCCACTAGTAACCCATTAAAGAAATGCTTC-3′ and 5′-CCCCTCGAGATTGAAACGCTCAACGCCTA-3′.

### Online supplemental material

Fig. S1 shows CoIP of Wdr92 with R2TP subunits, supplemental to Fig. 4. Table S1 shows a list of ciliary motility proteins in *Drosophila*. Table S2 shows abundance changes in motile cilia proteins in Wdr92 mutant testes: label-free quantitation intensities. Table S3 shows characteristics of R2TP/prefoldin-like genes in *Drosophila*. Table S4 shows RNAi lines and primers used in this study.

## Supplementary Material

Supplemental Materials (PDF)

Table S4 (Excel)
