# Supplementary material for: Ciliary dynein motor preassembly is regulated by Wdr92 in association with HSP90 co-chaperone, R2TP
Source: J Cell Biol. 2018 Jul 2;217(7):2583–98. doi: 10.1083/jcb.201709026 (PMC6028525; doi:10.1083/jcb.201709026)
Supplement: Supplemental Materials (PDF) [file JCB_201709026_sm.pdf]

Supplemental material

zur Lage et al., https://doi.org/10.1083/jcb.201709026

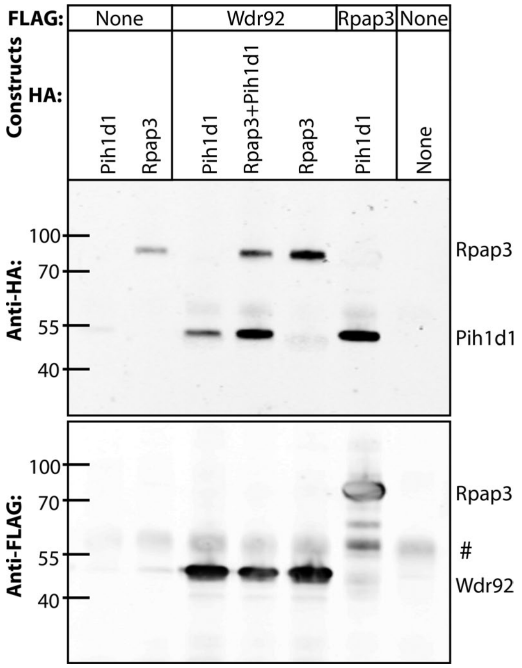

Figure S1. ***Drosophila*** **Wdr92 interacts with Rpap3 (Spaghetti) and Pih1d1 (CG5792) in S2 cells.** S2 cells were transfected with expression constructs for FLAG- and/or HA-tagged proteins as indicated. Complexes were immunoprecipitated with anti-FLAG antibodies and the Western blot probed with anti-HA and anti-FLAG antibodies. # indicates a common nonspecific band.

Table S1. **Ciliary motility proteins in *Drosophila***

Gene	Protein accession number	Human	Notes	Detected in spermatocytes[a]
**Dynein heavy chain**				
kl-3	A8Y5B7	DNAH8	ODA, sperm only	↓
CG9492	Q9VH97	DNAH5	ODA, Ch neuron only	
Dhc93AB	Q9VDG0	DNAH11	ODA, Ch neuron only	
CG3339	Q0KI05	DNAH9	ODA	
kl-5	Q5LJN5	DNAH17	ODA, sperm only	↓
Dhc98D	E1JJ04	DNAH10	IDA, dimeric	↓
kl-2	Q5LJP0	DNAH2	IDA, dimeric	↓
Dhc16F	Q9VWZ3	DNAH6	IDA, monomeric	↓
Dhc62B	Q7KVA7	DNAH12	IDA, monomeric	
Dhc36C	Q9VJC6	DNAH7	IDA, monomeric	↓
CG17150	Q9VZ77	DNAH3	IDA, monomeric	
**Dynein intermediate chain**				
CG9313	Q8MSJ9	DNAI1	ODA	
CG6053	Q9VTM3	DNAI2	ODA	
CG10859	Q9VJY4	DNAI2	ODA	nc
CG1571	Q9W3L0	DNAI2	ODA	
CG14838	Q9VS90	WDR63	IDA	↓
CG13930	Q9W0A6	WDR78	IDA, Ch neuron only	
Dic61B	Q9W0U9	WDR78	IDA	↓
CG15373	Q9VWZ4	LAS1	IDA	
CG31802	Q8T415	CENTRIN	IDA	nc
CG6971	Q9VGG6	DNALI1	IDA	↓
**Dynein light chain**				
CG7276	Q7KUM9			
CG5359	Q9VH45	DYNLT2		
roadblock	Q7KMS3	DYNLRB1		nc
robls54B	Q6IG91	DYNLRB2		
CG8800	A1Z7U2	DNAL1		
CG18130	Q9VYR5	NME8		nc
CG8407	A1Z8T9			nc
Dlc90F	Q94524	TCTEX-1		
**Docking adaptors**				
CG13202	A1Z8J9	CCDC103		
**ODA docking complex**				
CG14905	Q9VES4	CCDC63		
CG17083	Q9VCN4	CCDC114		↓
gudu	Q9VM21	ARMC4		↓
CG13502	D6W4X6	TTC25		
**ODA5/8/10 late assembly complex**				
CG14127	Q9VTQ3	CCDC151		
CG14185	Q9VW77	LRRC56		
**Nexin link/dynein regulatory complex**				
CG10958	Q9W3J8	DRC1/CCDC164		

Table S1. **Ciliary motility proteins in *Drosophila* (Continued)**

Gene	Protein accession number	Human	Notes	Detected in spermatocytes[a]
CG30259	Q9W212	CCDC65		
CG13125	Q9VL65	LRRC48		
CG14271	Q8MT08	GAS8		
CG14325	Q9VEH4	TCTE1		
CG8272	Q7K0V7	FBXL13		
lost boys	Q4V516	DRC7		nc
CG11041	A1ZBR3	EFCAB2		
CG13972	Q9VB28	IQCG		
CG13168	A1Z8V5	IQCD		
CG16789	Q9VHB4	IQCA		
**96-nm molecular ruler**				
CG17387	Q9VN57	CCDC39		nc
l(2)41ab	A0A0B7P7M6	CCDC40		nc
**Radial spokes**				
CG32392	Q9VRY7	RSPH3/AKAP		
CG5458	Q9VK29	RSPH1		nc
CG31803	Q8T3V7	RSPH9		nc
CG3121	Q9W1D3	RSPH4A		nc
TpnC41C	P47947	CALML5		
CG10014	Q9VGC1	ROPN1L		
CG17266	Q4V5H1	PPIL6		
CG8336	Q9VT21	PPIL6		
CG13501	Q9W2A3	RTDR1		
CG5001	Q9VPY9	DNAJB13		
Dnaj-1	Q53ZT0	DNAJB13		
CG15547	Q9VA28	NME5		
**Modifier of inner arms complex**				
CG17564	Q9VIY3	CCDC43A/B		
CG10750	Q9VIY1	CCDC43B		
**Calmodulin and spoke-associated complex**				
CG30275/268	Q8MME7	C20orf26		
CG15143/44	Q9VJC5	MAATS1		
**Protofilament stability and IDA docking**				
Tektin-C	Q8T3Z0	TEKT1		↓
Tektin-A	Q9V3M9	TEKT4		nc
CG3085	Q9W1V2	TEKT2		↓
CG17450	Q8IRZ1	TEKT3/5		
**Dynein preassembly factors and related cochaperones**				
dtr	Q8INT5	DNAAF1/LRRC50		nc
Nop17l	Q0E9G3	DNAAF2/KTU		↑
spaghetti	Q9V3E9	RPAP3		
CG5792	Q9VK58	PIH1D1		nc
CG5048	Q9VUG3	PIH1D3		nc
CG4022	Q9VSY1	MOT48?		

Table S1. **Ciliary motility proteins in *Drosophila* (Continued)**

Gene	Protein accession number	Human	Notes	Detected in spermatocytes[a]
CG17669	A1ZB91	DNAAF3		nc
CG14921	Q9VKJ5	DNAAF4/DYX1C1		↑
tilB	Q9VR52	LRRC6		nc
CG11253	Q9VU41	ZMYND10		nc
CG31320	Q8INF7	HEATR2		↑
reptin	Q9V3K3	REPTIN/RUVB2		↑
pontin	Q9VH07	PONTIN/RUVB1		
CG18675	Q9VZH1	C21orf59/Kurly		↑
CG18472	Q9VBA1	SPAG1		↓
**Others and human motile ciliopathy homologues**				
CG10064	Q9VS00	WDR16		↓
CG17230	Q7K4X4	CCDC11/CFAP53		nc
Mhc/zip	Q99323	MHC		
CG17687	Q9VU57	CFAP43		nc

List of predicted ciliary motility protein homologues in *Drosophila* compiled from literature review of other organisms (notably *Chlamydomonas*, human, and mouse) and use of orthologue search tool DIOPT (http://www.flyrnai.org/diopt; unpublished data).
[a]Detected in pupal spermatocytes by AP-MS in this study. nc = detected and no change in *Wdr92* mutant; ↓ = detected and reduced in mutant; ↑ = detected and increased in mutant. Quantitation data are shown in Table S2.

Table S2. **Abundance changes of motile cilia genes in Wdr92 mutant testes: Label-free quantitation intensities**

Genes	Label-free quantitation intensity										Ratio control/ mutant	P-value Student's *t*
	Control_1	Control_2	Control_3	Control_4	Control_5	Mutant_1	Mutant_2	Mutant_3	Mutant_4	Mutant_5		
Dhc98D	7.30E+08	7.13E+08	6.3E+08	7.51E+08	7.82E+08	1.83E+08	2.45E+08	2.1E+08	1.57E+08	2.4E+08	−1.817336	7.39E−07
Nop17l	2.72E+08	2.33E+08	2.55E+08	2.09E+08	2.34E+08	4.41E+08	5.07E+08	4.65E+08	4.58E+08	5.1E+08	0.985800	1.27E−06
kl-5	1.64E+09	1.41E+09	1.41E+09	1.63E+09	1.68E+09	1.01E+09	8.64E+08	1.18E+09	8.77E+08	8.4E+08	−0.710767	1.77E−04
gudu	1.21E+08	83194799	97437245	1.43E+08	1.18E+08	62768178	62483849	58905132	65879214	4.7E+07	−0.904065	4.55E−04
Dic61B	1.74E+08	1.43E+08	1.24E+08	1.66E+08	1.15E+08	77280977	92621899	77591216	69801756	9.3E+07	−0.803708	4.66E−04
Dhc16F	4.35E+07	45463014	59456038	69063016	64460856	34071922	32640957	30950084	21196019	2.1E+07	−1.018261	1.09E−03
Dhc36C	3.26E+08	2.24E+08	2.43E+08	2.89E+08	3.32E+08	1.4E+08	1.32E+08	1.95E+08	1.57E+08	9.7E+07	−0.992126	1.09E−03
CG3085	1.64E+08	1.41E+08	97740287	1.29E+08	1.18E+08	88639721	84596814	66048845	64681957	8.3E+07	−0.740690	1.56E−03
kl-3	5.74E+08	4.36E+08	3.79E+08	4.75E+08	4.93E+08	2.45E+08	2.41E+08	3.38E+08	3.41E+08	2.2E+08	−0.781947	1.60E−03
CG6971	3.10E+08	2.82E+08	3.22E+08	3.01E+08	3.63E+08	1.98E+08	2.51E+08	2.01E+08	2.7E+08	2.4E+08	−0.443519	3.40E−03
CG10064	1.02E+09	9.87E+08	8.05E+08	9.69E+08	9.53E+08	7.35E+08	8.41E+08	8.14E+08	8.17E+08	7.5E+08	−0.256317	6.80E−03
Tektin-C	1.64E+08	1.25E+08	93493323	1.53E+08	1.63E+08	77148245	92362891	72019787	1.06E+08	9E+07	−0.656659	7.14E−03
CG14838	9.65E+07	62749908	1.04E+08	87721066	99121178	62274146	53700046	75621860	34092948	4.6E+07	−0.756698	1.23E−02
CG14921	NaN	23969060	41612972	26700992	30525890	67445086	48045900	52958172	46226923	3.6E+07	0.934950	1.45E−02
CG31320	1.41E+09	1.41E+09	1.69E+09	1.56E+09	1.65E+09	1.88E+09	1.79E+09	2.08E+09	1.7E+09	1.7E+09	0.239790	1.60E−02
CG18675	7.24E+08	8.9E+08	6.77E+08	7.38E+08	8.07E+08	1.03E+09	1.08E+09	1.1E+09	8.32E+08	8.2E+08	0.336570	1.84E−02
CG17083	1.23E+08	71397984	74233137	1.1E+08	1.03E+08	57598983	64826035	75284003	62077194	7.5E+07	−0.501703	2.07E−02
reptin	3.61E+09	3.64E+09	4.48E+09	3.65E+09	4.39E+09	4.25E+09	4.58E+09	4.69E+09	4.57E+09	4.6E+09	0.208200	2.41E−02
kl-2	7.91E+07	52714993	1.44E+08	94103823	1.03E+08	55465986	52651088	74791967	54476852	4.8E+07	−0.673463	3.31E−02
CG18472/ Spag1	1.36E+09	1.28E+09	1.17E+09	9.83E+08	1.15E+09	9.34E+08	1.11E+09	9.01E+08	9.36E+08	1.1E+09	−0.255430	3.46E−02
Pih1D1	4.50E+08	4.88E+08	3.8E+08	4.65E+08	5.36E+08	3.97E+08	4.18E+08	4.02E+08	3.36E+08	4.2E+08	−0.225289	5.71E−02
Tektin-A	3.55E+08	2.2E+08	1.47E+08	2.48E+08	2.57E+08	1.48E+08	1.46E+08	1.99E+08	2.03E+08	1.5E+08	−0.503440	6.30E−02
CG3121	5.51E+08	3.27E+08	2.66E+08	4.58E+08	4.32E+08	2.9E+08	3.34E+08	3.34E+08	3.2E+08	2.8E+08	−0.340770	1.16E−01
CG31802	7.39E+07	1.17E+08	95005651	1.72E+08	1.04E+08	1.12E+08	56189997	1.1E+08	68566949	5.2E+07	−0.510934	1.37E−01
pont	3.51E+09	2.68E+09	4.13E+09	3.15E+09	3.26E+09	3.31E+09	4.28E+09	3.98E+09	4.38E+09	3.1E+09	0.188630	2.22E−01
dtr	3.79E+09	3.65E+09	3.37E+09	3.85E+09	3.71E+09	3.72E+09	3.68E+09	3.96E+09	3.63E+09	4.1E+09	0.056900	2.71E−01
CG17669	6.88E+08	8.99E+08	5.68E+08	6.49E+08	6.9E+08	6.34E+08	6.63E+08	5.37E+08	6.2E+08	7E+08	−0.138122	3.00E−01
CG10859	1.24E+08	1.47E+08	1.18E+08	1.44E+08	1.46E+08	1.32E+08	1.31E+08	1.33E+08	1.37E+08	1E+08	−0.101938	3.56E−01
l(2)41Ab	1.15E+08	93951965	1.01E+08	93187299	1.11E+08	1.13E+08	1.28E+08	1.12E+08	88853791	1.1E+08	0.094740	3.93E−01
CG8407-RA	9.51E+06	7371721	29311023	13356975	19257005	20388043	26725989	12588997	28365029	1.1E+07	0.391070	4.17E−01
CG5458	1.87E+08	1.61E+08	1.26E+08	1.46E+08	1.13E+08	1.33E+08	1.5E+08	1.26E+08	1.65E+08	9.6E+07	−0.131665	4.97E−01
CG17387-RA	9.13E+07	86652017	83273262	71293144	83108920	69358050	90094768	92112269	92343687	9.1E+07	0.062590	5.48E−01
CG11253	8.30E+08	9.3E+08	1.2E+09	8.05E+08	9.67E+08	7.84E+08	1.19E+09	9.35E+08	1.09E+09	9.7E+08	0.069680	6.40E−01
CG31803	2.47E+08	1.02E+08	1.59E+08	1.73E+08	2.33E+08	1.65E+08	2.14E+08	1.5E+08	1.87E+08	1E+08	−0.131660	6.62E−01
robl	1.91E+08	1.94E+08	1.1E+08	1.78E+08	1.58E+08	1.4E+08	1.67E+08	1.4E+08	1.33E+08	2.1E+08	−0.067306	7.34E−01
CG15547	7.19E+07	69926211	46538133	NaN	53658002	56778051	55651992	62379991	45306038	3.9E+07	0.108330	7.76E−01
Pih1D3	8.34E+08	7.51E+08	6.3E+08	5.8E+08	8.1E+08	5.92E+08	8.16E+08	6.4E+08	7.22E+08	9.5E+08	0.040570	8.07E−01
tilB	2.13E+08	1.49E+08	1.71E+08	1.95E+08	1.71E+08	1.78E+08	2.09E+08	1.86E+08	1.57E+08	1.7E+08	0.011540	9.19E−01
lobo	5.70E+07	51429044	66641152	96723962	87222076	60129099	93310106	78476099	56081826	6.2E+07	−0.021301	9.26E−01
CG17230	1.28E+08	1.11E+08	1.09E+08	91028817	1.32E+08	93299758	1.25E+08	1.05E+08	1.23E+08	1.2E+08	−0.008050	9.50E−01

Table S3. **Characteristics of R2TP/prefoldin-like genes in *Drosophila***

Protein	*Drosophila* gene	Ch neuron transcriptome[a]	Testis expression (FlyAtlas)	Wdr92 interactor (this study)
WDR92	*CG14353*	4.10	High, specific	not applicable
**R2TP**				
RPAP3	*spaghetti*	1.88	Moderate, general	+
PIH1D1	*CG5792*	−1.01	Moderate, general	+
RUVBL1	*pontin*	1.58	Moderate, general	+[b]
RUVBL2	*reptin*	2.53	High, specific	+[b]
**Prefoldin-like**				
PFDN2	*l(3)01239*	−2.03	Moderate, general	+
PFDN6	*CG7770*	−1.23	High, general	+
URI	*CG11416*	−1.28	Low, general	+
UXT	*l(2)35Cc*	Not determined	Low, general	+
PDRG1	*CG15863*	1.31	Low, general	+
**Chaperones**				
HSP90	*hsp83*	1.40	Very high, general	−
HSP70		All about threefold	Not determined	−

[a]Fold enrichment in Ch neuron precursor cells versus the rest of the embryo. Transcriptome data obtained as described previously (Cachero et al., 2011), but from a later time point (stage 13 embryos; unpublished data).
[b]In S2 cell coIPs.

Provided online is Table S4, in Excel, showing RNAi lines and primers used in this study.

Reference

Cachero, S., T.I. Simpson, P.I. Zur Lage, L. Ma, F.G. Newton, E.E. Holohan, J.D. Armstrong, and A.P. Jarman. 2011. The gene regulatory cascade linking proneural specification with differentiation in Drosophila sensory neurons. *PLoS Biol.* 9:e1000568 http://www.ncbi.nlm.nih.gov/pmc/articles/PMC3023811/. https://doi.org/10.1371/journal.pbio.1000568
